# Apoptotic vesicles resist oxidative damage in noise-induced hearing loss through activation of FOXO3a-SOD2 pathway

**DOI:** 10.1186/s13287-023-03314-7

**Published:** 2023-04-15

**Authors:** Xiaotong Huang, Xiaoxing Kou, Ting Zhan, Guokun Wei, Feinan He, Xueli Mao, Haidi Yang

**Affiliations:** 1 Department of Otolaryngology, Sun Yat-Sen Memorial Hospital, Sun Yat-Sen University, Guangzhou, 510120 China; 2 Guangdong Provincial Key Laboratory of Malignant Tumor Epigenetics and Gene Regulation, Medical Research Center, Sun Yat-Sen Memorial Hospital, Sun Yat-Sen University, Guangzhou, 510120 China; 3 Hospital of Stomatology, Guanghua School of Stomatology, South China Center of Craniofacial Stem Cell Research, Guangdong Provincial Key Laboratory of Stomatology, Sun Yat-Sen University, Guangzhou, 510055 China; 4 Key Laboratory of Stem Cells and Tissue Engineering, Ministry of Education, Sun Yat-Sen University, Guangzhou, 510080 China; 5 Department of Otolaryngology, Zhujiang Hospital of Southern Medical University, Southern Medical University, Guangzhou, 510285 China; 6 Department of Otorhinolaryngology Head and Neck Surgery, The Second Hospital of Jilin University, Changchun, 130041 China; 7 Department of Hearing and Speech Science, Guangzhou Xinhua University, Guangzhou, 510310 China

**Keywords:** Mesenchymal stem cell, Apoptotic vesicles, Oxidative stress, Noise-induced hearing loss, Mitochondrial superoxide dismutase 2 (SOD2)

## Abstract

**Background:**

Mesenchymal stem cell (MSC) transplantation is a promising therapeutic approach for noise-induced hearing loss (NIHL). As the indispensable role of apoptosis in MSC transplantation was raised, the benefits of MSC-derived apoptotic vesicles (apoVs) in several disease models have been proved. However, whether apoVs benefit in NIHL have not been studied yet.

**Methods:**

Female CBA/J mice and HEI-OC1 cells were used in this study. Flow cytometry, nanoparticle tracking analysis (NTA) and transmission electron microscopy (TEM) were used to characterize apoVs. Proteomic analysis was used to identify function proteins in apoVs. Immunofluorescence was used to reveal distribution pattern. Auditory brainstem response (ABR) test was used to measure the effect of apoVs treatment. DCFH-DA staining and MitoSOX staining were used to indicate oxidative damage. Western-blot and qRT-PCR were used to study the signaling pathways.

**Results:**

We found that apoVs can be endocytosed by hair cells through systemic administration. Importantly, apoVs administration effectively attenuated NIHL and reduced hair cell loss by resisting oxidative damage in vivo. Further, apoVs application activated forkhead box o3 (FOXO3a)—mitochondrial superoxide dismutase 2(SOD2) pathway, which may relate to signal transduction and activators of transcription 3 (STAT3) in apoVs.

**Conclusions:**

These findings uncovered the role of apoVs in preventing NIHL and resisting oxidative damage, indicating that apoVs is a promising way for inner ear delivery and a prospective cell-free therapy for NIHL.

**Supplementary Information:**

The online version contains supplementary material available at 10.1186/s13287-023-03314-7.

## Background

Noise-induced hearing loss (NIHL) is a major health issue worldwide. According to Global Burden of Disease study 2019 (GBD2019), about 5% (1,456,662,173 people) of the world’s population is suffering from hearing impairment [1], with a years lived with disability (YLDs) of 43.45 million, among which 7 million were attributable to occupational noise exposure [2]. Unlike the few progress in drug therapy, mesenchymal stem cell (MSC) transplantation was proved effective in multiple sensorineural hearing loss [3–7], but the mechanism behind remained unclear. As traditional acknowledgment, the effect of MSCs based on their ability of homing [8] and multi-differentiation to replace the injured cells. Although Sevda Pouraghaei et al. surprisingly found that MSCs can differentiate into auditory progenitor cells in vitro [9], in vivo evidence doubted that stem cells expressed auditory hair cell markers without auditory improvements [10], which greatly challenged the classical theory.

Current studies pointed out that the therapeutic effect of MSCs was based on their secretion of extracellular vesicles which contained functional proteins, microRNAs, small nucleolar RNAs, transfer RNAs, etc. [11–13]. Interestingly, MSCs underwent apoptosis after systemic administration [14], which was essential for the therapeutic effect of MSCs transplantation [15]. During apoptosis, MSCs released numbers of extracellular apoptotic vesicles (apoVs), including apoptotic exosomes (< 150 nm in diameter), apoptotic microvesicles (0.1–1 μm in diameter) and apoptotic bodies (1–5 μm in diameter) [16]. Previous studies proved that the application of MSC-derived apoVs can ameliorate osteopenia [17], myocardial infarction [18] and type 2 diabetes [19], indicating that apoVs therapy may be a prospective cell-free therapy. But their benefits in NIHL remained unknown.

Permanent elevation of hearing threshold and loss of hair cells (HCs) are the main feature of NIHL [20], which are mostly due to reactive oxygen species (ROS) accumulation including free radicals superoxide (O_2_^·−^), hydroxyl radical and singlet oxygen. The level of ROS elevated in 1–2 h after noise exposure [21] and persisted for 7–10 days [22]. On the one hand, ROS induced the formation of lipid peroxidation products and led to hair cell apoptosis [23]. On the other hand, ROS can also promote the production of pro-inflammatory cytokines, which cause further damage [24]. One of the reasons for ROS accumulation is a lower activity of oxidoreductases. Mitochondrial superoxide dismutase 2 (SOD2) is an important oxidoreductase in maintaining ROS level in the mitochondrial matrix, by catalyzing O_2_^·−^ derived from the electron transport chain into hydrogen peroxide (H_2_O_2_) for further elimination by catalase [25]. The disturbance, suppression or deletion of SOD2 may lead to severe oxidative damage [26]. Clinical studies found that SOD2 genetic polymorphisms related to the susceptibility of NIHL [27, 28]. After noise exposure, activity of SOD2 reduced [29], and upregulation of SOD2 can resist oxidative stress thus protecting NIHL [30, 31]. Although studies have indicated that SOD2 plays a role in MSC therapy to resist oxidative damage [32–34], the relationship between MSC-derived apoVs and oxidative damage or SOD2 in NIHL has not been studied.

In this study, we aimed to examine the effect of apoVs treatment on NIHL and its potential mechanism against oxidative stress. We characterized proteins in MSC-derived apoVs and uncovered a potential relationship between apoVs and oxidative stress. We proved that apoVs can pass through blood–labyrinth barrier and be endocytosed by hair cells. Application of apoVs successfully prevented NIHL through resisting oxidative damage and reduced mitochondrial ROS levels. Mechanically, apoVs promoted FOXO3a nuclear translocation and activated SOD2 expression, which may be related to STAT3 in apoVs. Overall, our findings indicated that MSC-derived apoVs showed great benefits of inner ear delivery and oxidative damage resistance in NIHL.

## Methods

### Animals and study design

In this study, CBA/J mice were used to establish NIHL model with a 2-h noise exposure, which was used in previous studies and proved successful to induce permanent hearing loss [35, 36]. Healthy female CBA/J mice (brought from Beijing HFK Bioscience Company) at 8 weeks of age were used in experiments. Animal experiments were conducted in the Laboratory Animal Center, Sun Yat-Sen University. Mice were raised under specific pathogen-free (SPF) conditions in an environment of 24 °C, 12-h light/dark cycles and 50% humidity with abundant food and water.

The reporting of animal experiments adhered to the ARRIVE guidelines. The protocol was prepared before the study without registered. Sample size was determined under the consideration of cost and time consumption. Because there is no similar experiment for reference, we did not perform a priori sample size calculation. Mice with baseline ABR threshold over 40 dB were excluded after baseline ABR tests because we considered they have experienced hearing loss. In addition, no mice were excluded from analysis. A single mouse was used as an experiment unit. Mice were killed with overdose of ketamine and xylazine through intraperitoneal injection. No adverse events were seen in experiments.

### Isolation, culture and characterization of MSCs

Human umbilical cords were kindly provided by Department of gynecology and obstetrics, The Sixth Affiliated Hospital, Sun Yat-Sen University. The isolation of umbilical mesenchymal stem cells (UMSCs) was described as previous studies [37]. MSCs were cultured in α-Minimum Essential Medium (α-MEM) (BI, #01-042-1ACS) supplemented with 10% fetal bovine serum (FBS) (GIBCO, #10270-106) at 37 °C and 5% CO_2_. For passage, the adherent cells were digested with TrypLE (GIBCO, #12605-028) and harvested through centrifugation at 1500 rpm for 5 min. MSCs at the eight to ten passages were used for experiments.

MSC surface markers were characterized by flow cytometric analysis. MSCs were harvested and suspended in phosphate-buffered saline (PBS) at a density of 4 × 10^6^ cells/ml. Then, MSCs suspension was incubated with anti-CD90 (PE-conjugated, BD Biosciences, #555596, 1:100), anti-CD105 (FITC-conjugated, BD Biosciences, #561443, 1:20), anti-CD73 (APC-conjugated, BD Biosciences, #560847, 1:20), anti-CD45 (BV510-conjugated, BD Biosciences, #563204, 1:20), anti-CD34 (BV480-conjugated, BD Biosciences, #746688, 1:100) and anti-CD11b (PE/Cy7-conjugated, BD Biosciences, #557743, 1:20) at 4 °C for 30 min. BD FACS Verse flow cytometer(BD Biosciences) and FlowJo 10.6.2 software were used to detect and analyze their fluorescence signals. For colony formation, MSCs were seeded at a density of 2 × 10^3^ cells/dish and cultured for 14 days before fixation with 4% paraformaldehyde (PFA) (MACKLIN, #30525-89-4) and staining with crystal violet (Beyotime, #C0121). Inverted microscope (Olympus IX73) was used to screen for colonies with over 50 cells. For multiple differentiation, MSCs were cultured in osteogenic medium (α-MEM with 10%FBS containing 50 μg/ml ascorbic acid, 10 mM β-glycerophosphate and 10 nM dexamethasone) for 21 days. Then, alizarin red S (Beyotime, #C0140) staining was performed to detect osteogenesis. Besides, MSCs were cultured in adipogenic medium (ExCell Bio, #MD000-N011) for 25 days before oil red O (ORO) (Beyotime, #C0158S) staining to detect adipogenesis. MSCs without induction also underwent alizarin red S and ORO staining as a negative control. Images were taken using inverted microscope (Olympus IX73).

### Collection and identification of apoVs

MSCs were cultured with α-MEM containing 500 nmol/L staurosporine (STS, CST, # 9953S). After 9 h of treatment, microscope was used to confirm the apoptotic morphology of MSCs. ApoVs were collected according to previous studies [19]. In brief, the culture supernatant of apoptotic MSCs was collected and centrifuged at 800 g for 10 min and 2,000 g for 10 min in succession. Then, supernatant was collected and centrifuged at 16,000 g at 4 °C for 30 min. Then, pellet apoVs were resuspended with filtered PBS. Finally, BCA Protein Assay Kit (CoWin Biosciences, #CW0014S) was used to quantify protein concentration of apoVs.

For transmission electron microscope (TEM), apoVs were suspended in filter PBS. Twenty microliters of apoVs suspension was added to formvar-coated copper grids. Then, uranyl acetate was incubated with apoVs suspension for 3 min. Excessive suspension was removed with a filter paper after every step. Samples were dried for 5 min at room temperature before imaging with JEM-1200EX TEM (JEOL) operating at 100kv.

Nanoparticle tracking analysis (NTA) was performed to characterize the size distribution of apoVs. ApoVs were suspended in filter PBS for capturing with NanoSight NS300 (Malvern) with 405 nm laser and sCMOS camera. Data were analyzed by NTA 3.2 Dev Build 3.2.16 software (Malvern, UK).

For apoptosis-related protein detection, protein from MSCs and apoVs was extracted and quantified. Apoptosis-related proteins were detected by western blotting using anti-Caspase-3 (ImmunoWay, #YT5204, 1:1000), anti-cleaved-Caspase-3 (ImmunoWay, #YC0006, 1:1000) and anti-β-actin (Ray, #RM2001, 1:10,000) antibodies. Detailed protocols were stated below.

### Proteomic analysis

For proteomic analysis, total protein of apoVs was extracted and lysed. LC–MS/MS analysis was conducted on Easy-nLC 1200 system (Thermo Scientific) and Q-Exactive Plus mass spectrometer (Thermo Scientific). MaxQuant 1.6.1.0 software was used to analyze raw data, and Uniprot Protein Database with false discovery rate (FDR) set at 0.01 was used to identify proteins and peptides. All the proteins we identified are listed in Additional file 1: Table S1. They were further analyzed based on Gene Ontology (GO), Kyoto Encyclopedia of Genes and Genomes (KEGG) databases and the PANTHER classification system.

### ApoVs distribution after intravenous administration

Extracted apoVs were labeled with PKH26 fluorescence using PKH26 Red Fluorescent Cell Linker Mini Kit (Sigma, MINI26-1KT) according to manufacture instruction. In brief, apoVs were incubated with PKH26 staining solution for 5 min in the dark. Labeled-apoVs were harvested from centrifugation and washed with PBS three times to remove excess dye. After the last centrifugation, the pellet apoVs were suspended with filtered PBS and the supernatant was collected as the negative control. Six CBA/J mice were divided into 6 groups (control, apoVs at 1 h, 2 h, 4 h, 8 h and 15 h group) randomly using random number generator. Mice were injected with suspended apoVs (100ug in 200ul PBS) or equal volume of negative control through tail vein. To examine its distribution at different time points, mice from apoVs group were killed at each time point (1 h, 2 h, 4 h, 8 h and 15 h after injection). Cochlea, liver, lung and kidney were harvested and fixed in 4% PFA overnight.

Cochlea surface preparation was performed as our previous study [35]. Briefly, after fixation, cochleae were decalcified with 4% sodium ethylenediaminetetraacetic acid (EDTA) for 2 days. Basilar membranes were micro-dissected from cochlea and divided into three turns (apex, middle and base). After permeabilized with 2.5% Triton X-100 solution for 20 min and blocked with 10% normal goat serum for 30 min, basilar membranes were incubated with YF488-conjugated phalloidin (UElandy, #YP0059S, 1:200) overnight at 4 °C and DAPI (Solarbio, #C0065) for 10 min. Between every step, basilar membranes were washed with PBS for 5 min three times. All steps above were performed in dark. After the last wash, basilar membranes were carefully transferred to slide glasses and covered with mounting medium (Solarbio, #S2100) before placing a cover glass. Samples were photographed with a 63×-magnification lens under confocal microscopy (Zeiss LSM 710).

Livers, lungs, kidneys and some cochleae were prepared for frozen section. After fixation, livers, lungs and kidneys went through gradient dehydration with 15% sucrose for 24 h and 30% sucrose for another 24 h. Notably, cochleae were decalcified with 4% EDTA for 14 days before gradient dehydration. Then, tissues were embedded in O.C.T compound (Sakura, #4583) and cryosectioned at a thickness of 10 μm for cochlea section and 5 μm for liver, lung and kidney section using a freezing microtome (Leica CM1450). Cochlea sections were then incubated with phalloidin and DAPI after permeabilization and blocking as above. Liver, lung and kidney sections were incubated with DAPI directly. Samples were photographed with a 10×-magnification lens under confocal microscopy.

For blinding, 2 investigators were involved in animal experiment. One investigator (TZ) was aware of the group allocation and responsible for apoVs administration. Another investigator (XH) was responsible for sample processing and statistical analysis and was blind for the group allocation.

### Measurement of auditory brainstem response (ABR)

At baseline and 2 weeks, 4 weeks, 6 weeks, 8 weeks after noise exposure, we performed ABR measurements on CBA/J mice using Tucker Davis Technology (TDT) System III hardware and software (Alachua, FL, USA) as our previous study [35]. Mice were anesthetized with ketamine and xylazine through intraperitoneal injection. Three subdermal needle electrodes were placed under the left ear, at the vertex, and in the middle of the back. Then, ABR was recorded at 8 kHz, 16 kHz and 32 kHz in the left ear of each animal with an average response of 1024 stimuli for each sound stimulus level reducing at 5 dB intervals from 100 dB sound pressure level (SPL). ABR threshold was defined as the lowest stimulation decibel level, which produced replicable waveforms. Threshold shift of each mouse was defined as the changes of ABR threshold after noise exposure.

### ApoVs, MSCs treatment and noise exposure

ApoVs were extracted, quantified with protein concentration and suspended in 200 ul PBS (100 μg per mouse) with 10 ul 0.2% heparin (STEMCELL Technologies, #07980). MSCs were harvested through digestion and centrifugation and suspended in 200 ul PBS (1 × 10^6^ cells per mouse) with 10 ul 0.2% heparin. Twenty-eight CBA/J mice were divided into 4 groups randomly using random number generator. Exactly 7 mice for Control, 6 for Noise, 7 for Noise + apoVs and 6 for Noise + UMSCs group after exclusion of 2 mice whose baseline ABR threshold were over 40 dB. CBA/J mice from Noise + apoVs or Noise + UMSCs group were injected with apoVs or MSCs through tail vein 2 h before noise exposure, while Noise and Control group were injected with equal volume of PBS with heparin at the same time.

For noise exposure, CBA/J mice were placed separately in cages and exposed to a band pass noise of 8 to 16 kHz at 110 dB SPL for two hours in a sound-proofed room. Sound-level meters were used to calibrate noise intensity before and after noise exposure to guarantee a 2-h stable noise exposure. Control mice were placed in the same cages without noise exposure for 2 h.

For blinding, two investigators were involved in animal experiment. One investigator (TZ) was aware of the group allocation and responsible for apoVs administration. Another investigator (XH) was responsible for ABR measurement, sample processing and statistical analysis and was blind for the group allocation.

### Bulla injection

ApoVs were extracted as above and suspended in 5 ul PBS. Twelve CBA/J mice were divided into 3 groups randomly using random number generator. Exactly 3 mice for Control, 4 for Noise and 3 for Noise + apoVs (bulla injection) after exclusion of 2 mice whose baseline ABR threshold were over 40 dB. After anesthetized with ketamine and xylazine, mice were placed left-lateral position. The surgical area was disinfected with iodophor and covered with sterile towel. Then, a 2-cm cut was made along the retroauricular groove. After separating subcutaneous tissue, the facial nerve and sternocleidomastoid muscle were exposed. The posterior wall of bulla can be exposed after separating sternocleidomastoid muscle. Then, an opening in the bulla was made by drilling with a 27-G needle and 5 μl apoVs suspension was injected with a microinjector. (Control and Noise group were injected with 5 μl PBS.) After injection, the opening was sealed with subcutaneous tissue and the incision was sewn up. Mice were placed left-lateral position until analepsia.

Investigators could not be blinded to the group allocation due to the operation scar. However, the ABR measurement was taken using TDT system to create waveforms and ABR threshold was strictly defined as the lowest stimulation decibel level, which produced replicable waveforms to reduce subjective effects.

### Immunohistochemistry for cochlear surface preparations

1 h and 1 day after noise exposure, CBA/J mice were killed. Their cochleae were dissected and immediately fixed in 4% PFA overnight. The protocol for cochlea surface preparation was stated above. After permeabilization and blocking, basilar membranes were incubated with anti-4-hydroxynonenal (4-HNE, Abcam, #ab46545, 1:200), anti-3-nitrotyrosine (3-NT, Abcam, #ab110282, 1:400) overnight at 4 °C and secondary antibody (CST, #8889, 1:400) for 1 h at room temperature. Then basilar membranes were incubated with phalloidin (YF488-conjugated, UElandy, #YP0059S, 1:200) for 2 h and DAPI for 10 min. Samples were photographed with 63x-magnification lens under confocal microscope.

Semi-quantification of fluorescence intensities of 4-HNE and 3-NT was analyzed using original confocal images, which is taken with the same 63×-magnification lens under identical settings. Using image J software, the intensity of the background was subtracted and the average grayscale intensity was measured to represent the fluorescence intensity of each image. For each individual, the relative fluorescence intensity was determined by normalizing the ratio to control.

### Hair cell (HC) counts and spiral ganglion cell counts

At 2 weeks and 8 weeks after noise exposure, CBA/J mice were killed after ABR measurements and cochleae were harvested. Basilar membranes samples were prepared as above and incubated with rabbit anti-mouse Myosin-VIIa (a marker for hair cell, Proteus Biosciences, #25–6790, 1:400), the corresponding secondary antibody, phalloidin and DAPI. Samples were imaged under fluorescence microscope (Olympus BX63). As previously described [35], outer hair cells (OHCs) were counted along the entire basilar membranes. The percentage of loss of OHCs in each 0.5 mm length of basilar membranes was calculated.

For spiral ganglion cell counts, samples for cochlea frozen section were prepared as above 8 weeks after noise exposure. Cochlea sections were then stained with hematoxylin–eosin (H&E). The images were taken under the microscope (Nikon Ni-U). Numbers of spiral ganglion cells were counted in every 1 mm × 1 mm area.

### Culture of HEI-OC1 cells and uptake of apoVs by HEI-OC1 cells in vitro

For in vitro experiments, we used HEI-OC1 cell, an auditory hair cell line from mouse, to simulate the changes of hair cells in different conditions. HEI-OC1 cells were cultured in high-glucose Dulbecco's modified Eagle medium (DMEM, GIBCO, #C11995500BT) supplemented with 10% FBS under the condition of 10% CO_2_ and 33 °C. For passage, the adherent cells were digested with 0.25% Trypsin–EDTA (GIBCO, #25,200–072) and harvested through centrifugation at 1,000 rpm for 5 min.

To examine the uptake of apoVs by HEI-OC1 cells, PKH26-labeled apoVs and negative supernatant from above were used. HEI-OC1 cells were seeded into a 24-well plate placed with 14-mm cover slip. After adherence, HEI-OC1 cells were washed with PBS twice and incubated with quantified apoVs or equal volume of negative supernatant, at a concentration of 5, 10, 20, 40, 60, 80, 100 ug/ul. 2, 3, 4, 6, 8, 10, 12, 24 h after incubation, apoVs and negative supernatant were removed. HEI-OC1 cells were washed with PBS twice and underwent fixation, permeabilization and blocking in succession. Then, HEI-OC1 cells were incubated with Myosin-VIIa overnight at 4 °C, the secondary antibody for 1 h and DAPI for 10 min. All steps above were performed in dark, and samples were washed with PBS for 5 min twice between each step. Finally, cover slips were taken and placed on slide glassed covered with mounting medium. Slides were observed and imaged under fluorescence microscope.

### ApoVs and hydrogen peroxide (H_2_O_2_) treatment of HEI-OC1 cells

For apoVs treatment, quantified apoVs without PKH26 label were used. HEI-OC1 cells were pretreated with 10 μg/μl apoVs for 48 h. To stimulus the oxidative stress that hair cells faced under noise exposure, HEI-OC1 cells were exposed to 1 mM H_2_O_2_ for 1 h as a suitable cell damage model.

### Cell vitality assay

Cultured HEI-OC1 cells were seeded in 96-well plates at a density of 2500 cells/well. After adherence, HEI-OC1 cells were treated with H_2_O_2_, with or without pretreatment of apoVs, as above. Then, culture medium was replaced by DMEM with 10% cell counting kit-8 reagent (CCK8, APExBIO, #K1018) solution. After a 2-h incubation at 37 °C, cell numbers were quantified as an absorbance of 450 nm using a Bio-Tek SYNERGY H1 microplate reader (Bio-Tek).

### Measurements of cellular reactive oxygen species (ROS) by flow cytometry

Cellular ROS level was measured by DCFH-DA staining (Beyotime, #S0033S) according to instruction. After H_2_O_2_ treatment, the culture medium of HEI − OC1 cells was replaced by DMEM containing DCFH-DA (10 μM) for 20 min at 37 °C. Then HEI-OC1 cells were washed with PBS twice and digested with 0.25% Trypsin–EDTA for 1 min. After centrifugation, HEI-OC1 cells were suspended with 200 μl PBS into a flow cytometry test tube (BD Biosciences, #352063). Fluorescent signals were detected by BD FACS Verse flow cytometer.

### Detection of mitochondrial ROS levels

Mitochondrial ROS levels were detected by MitoSOX (Invitrogen, #M36008), a fluorescent dye specifically targeting mitochondria superoxide in living cells. HEI-OC1 cells were seeded into a 24-well plate placed with 14 mm cover slip and treated with apoVs or H_2_O_2_. According to instruction, MitoSOX staining solution were diluted at 1:1000 with preheated Hank's Balanced Salt Solution (HBSS, GIBCO, # C14175500BT). After H_2_O_2_ treatment, HEI-OC1 cells were washed twice with preheated HBSS and incubated with MitoSOX staining solution for 10 min at 37 °C in the dark. Then HEI-OC1 cells successively underwent fixation, permeabilization, blocking and DAPI staining as above. Samples were observed and imaged with a 60×-magnification lens under fluorescence microscope.

### Preparation of cytoplasmic and nuclear extracts

Nuclear and Cytoplasmic Protein Extraction Kit (Beyotime, #P0027) was used to extract nuclear and cytoplasmic proteins from HEI-OC1 cells. According to instruction, HEI-OC1 cells were washed with PBS and transferred to microcentrifuge tubes. Then, samples were incubated with solution A for 15 min and solution B for 1 min. After full vortex, samples were centrifugated at 14,000 g for 5 min. The supernatant was collected as cytoplasmic extracts. The pellet was further suspended in Nuclear Protein Extraction Solution and incubated for 30 min, with vortex for 20 s frequently. Samples were centrifuged at 14,000 g for 10 min, and supernatant was collected as nuclear extracts. All steps above were performed on ice. Finally, cytoplasmic and nuclear extracts were stored at − 80 °C for further used in western blotting.

### Extract of total protein of HEI-OC1 cells and western blot assay

After apoVs and H_2_O_2_ treatment, the total protein of HEI-OC1 cells was extracted by RIPA lysis buffer (Beyotime, #P0013B) with 1% protease inhibitors (CoWin Biosciences, #CW2200S) and 1% phosphatase inhibitor (CoWin Biosciences, #CW2383S). Samples were then centrifuged at 14,000 g for 30 min at 4 °C. The supernatant was collected as total protein extracts. After the quantification of protein concentration by a BCA Protein Assay Kit (CoWin Biosciences, #CW0014S), protein samples were added with SDS-PAGE loading buffer (CoWin Biosciences, #CW0027S) and heated at 96 °C for 10 min for protein denaturation.

Western blot assays were performed as previous study [35]. Total, cytoplasmic or nuclear proteins (20 μg) isolated from HEI-OC1 cells underwent electrophoresis with a 12.5% Protein Gel. Then, proteins were transferred to a polyvinylidene fluoride (PVDF) membrane (Merck Millipore, #ISEQ00010) and blocked with 5% bovine serum albumin (BSA, MRC Biotechnology, #CCS30014.01) for 1 h. Afterward, the blocked membranes were separated and incubated with different primary antibodies: anti-GAPDH (Ray, #RM2002, 1:10,000), anti-β-actin (Ray, #RM2001, 1:10,000), anti-SOD2 (Proteintech, #66,474–1, 1:10,000), anti-FOXO3a (CST, #12,829, 1:1000), anti-STAT3 (Abcam, ab109085, 1:1000), anti- Phospho-STAT3 (Tyr705) (Affinity, # AF3295, 1:1000), anti-LaminB1 (Proteintech, #12,987–1-AP-50ul, 1:2000) overnight at 4 °C. Then, the membranes were incubated with secondary antibody for 1 h at room temperature. Between each step, membranes were washed twice by TBST (triethanolamine-buffered saline solution (TBS) with Tween-20) for 5 min. Signals were detected by Syngene G:BOX Chemi XT4 (Syngene) using a ECL chemiluminescent substrate kit (Fude Biological Technology, #FD8020) and analyzed by ImageJ software. Protein expression of total protein extracts was normalized to β-actin. Protein expression of cytoplasmic and nuclear protein extracts was normalized to GAPDH and LaminB1 respectively.

### RNA extraction and quantitative real-time polymerase chain reaction

mRNA of HEI-OC1 cells was extracted using EZ-press RNA Purification Kit (EZBioscience, #B0004DP). 1 μg of mRNA were reverse-transcribed to cDNA samples in a total volume of 20 μl using Color Reverse Transcription Kit (EZBioscience, #A0010CGQ). Primers were synthesized by the Beijing Genomics Institute (Shenzhen, China). Sequences used for amplifications were as followed: SOD2 (Forward, 5′-ACGCCACCGAGG AGAAGTACC-3′, Reverse, 5′- CGCTTGATAGCCTCCAGCAACTC-3′), glyceraldehyde- 3-phosphate dehydrogenase (GAPDH, Forward, 5′-TGAACGGGAAGCTCACTGG-3′, Reverse, 5′-GCTTCACCACCTTCTTGATGTC-3′). A color SYBR Green qPCR master mix (EZBioscience, #A0012-R2) was used to amplify cDNA samples, and the Bio-Rad CFX connect real-time PCR system (Bio-Rad) was used to detect the signals. GAPDH is used as internal reference gene, and the results were calculated with the formula:2^−ΔΔCt^.

### Immunocytochemistry of HEI-OC1 cells

HEI-OC1 cells were seeded into a 24-well plate placed with 14-mm cover slip. After H_2_O_2_ treatment, HEI-OC1 cells successively underwent fixation, permeabilization and blocking as above. Then, HEI-OC1 cells were incubated with anti-FOXO3a (CST, #12829, 1:200) overnight at 4 °C, secondary antibody (CST, #4412S, 1:400) for 1 h, YF594-conjugated phalloidin (UElandy, # YP0052S, 1:200) for 2 h and DAPI staining for 10 min. Samples were washed with PBS for 5 min twice between each step. Slides were observed and imaged under fluorescence microscope.

### Statistical analysis

Data were analyzed using SPSS Statistics 25 software. Comparisons between continuous variable of two groups were performed by two-tailed Student’s t-test. For multiple group comparisons, one-way ANOVA or Kruskal–Wallis H test with multiple comparisons was used depending on whether they obeyed normal distribution and homogeneity of variance. Values of *p* < 0.05 were considered significant.

## Results

### Characterization of MSCs and MSC-derived apoVs

According to minimal criteria for defining multipotent mesenchymal stromal cells stated by International Society for Cellular Therapy [38], we characterized MSCs before used in our study. Through flow cytometry, we found that MSCs highly expressed CD105, CD90 and CD73 while negatively expressed CD45, CD11b, and CD34 (Additional file 2: Fig. S1a). MSCs preserved the ability of proliferation such as colony formation (Additional file 2: Fig. S1b). Also, MSCs were able to differentiate into osteoblast or adipocyte after induction, as proved by alizarin red S and ORO staining (Additional file 2: Fig. S1c). As previous articles [19, 39], apoVs were collected from supernatant of staurosporine (STS)-induced apoptotic MSCs. After STS treatment, MSCs shrunk in size and suspended into culture medium (Additional file 2: Fig. S1d), representing that they were experiencing apoptosis. Then, we collected apoVs from gradient centrifugation (Additional file 2: Fig. S1e) and characterized their morphology, size distribution and presence of apoptosis-associated markers. From transmission electron microscope (TEM), apoVs presented an extracellular-vesicle-like shape (Additional file 2: Fig. S1f). Their diameters distributed from 100 to 600 nm through nanoparticle tracking analysis (NTA) (Figure S1g). Western blot showed that apoVs contained apoptosis-associated proteins like caspase-3 and cleaved caspase-3 (Additional file 2: Fig. S1h). The characteristics of apoVs were in agreement with previous studies [19, 39], suggesting the consistence and stabilization of apoVs.

### ApoVs from MSCs were enriched with various oxidoreductase

**Fig. 1:**
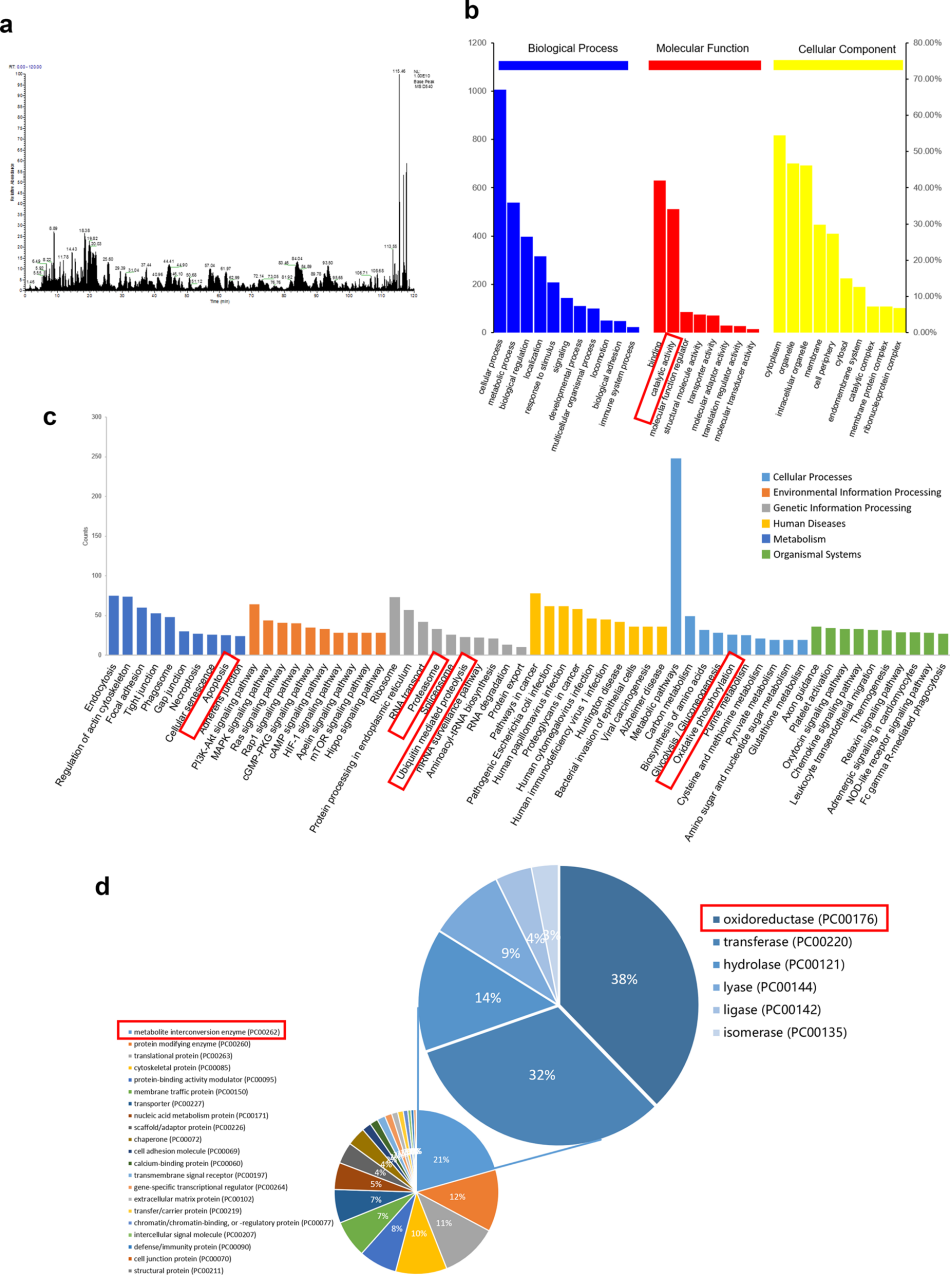
Proteomic analysis of MSCs-derived apoVs. **a** Base Peak Chromatogram of LC–MS/MS analysis. **b** GO analysis of identified apoVs proteins categorized as “Cellular component”, “Molecular function”, and “Biological process”. **c** KEGG pathway analysis of identified apoVs proteins. **d** The PANTHER classification of identified apoVs proteins

Previous studies stated that functional proteins in extracellular vesicles are the key to their therapeutical effect [40]. To further identify the proteins in MSCs-derived apoVs, we used shotgun proteomics techniques and identified 1781 proteins in total (Table 1, Fig. 1a). From GO database, the proteins we identified were divided into three domains including “biological process”, “molecular function” and “cellular component” to help analyze the information of their functions. Analysis revealed that most of the proteins in apoVs located in cytoplasm and membrane, served as a role for binding and catalytic activity and took part in cellular process such as metabolic process, biological regulation and localization (Fig. 1b), which was also proved by enrich analysis (Additional file 3: Fig. S2). KEGG analysis showed that proteins in apoVs were associated with “apoptosis” in “cellular processes” domain, “proteasome” and “ubiquitin mediated proteolysis” in “Genetic Information Processing” domain, and “oxidative phosphorylation” in “metabolism” domain (Fig. 1c). We then analyzed their classification according to the PANTHER classification system [41]. We found that the biggest class of proteins we identified is metabolite interconversion enzyme (21%), followed by protein-modifying enzyme (12%), translational protein (11%), cytoskeletal protein (10%), protein-binding activity modulator (8%), membrane traffic protein (7%) and transporter (7%). Within metabolite interconversion enzyme, 38% were oxidoreductase, 32% were transferase and the rest were hydrolase, lyase, ligase and isomerase (Fig. 1d). These results suggested that apoVs were enriched with oxidoreductase, which took part in the oxidative phosphorylation and helped balance oxidoreduction state.

**Table 1:** Statistics of characterized protein and peptides from proteomics analysis

Sample	Protein groups	Peptides	PSM
Apoptotic vesicles (apoVs) from mesenchymal stem cells (MSCs)	1781	8910	10,852

### ApoVs distributed in several parts of cochlea as well as liver, lung and kidney after systemic administration

**Fig. 2:**
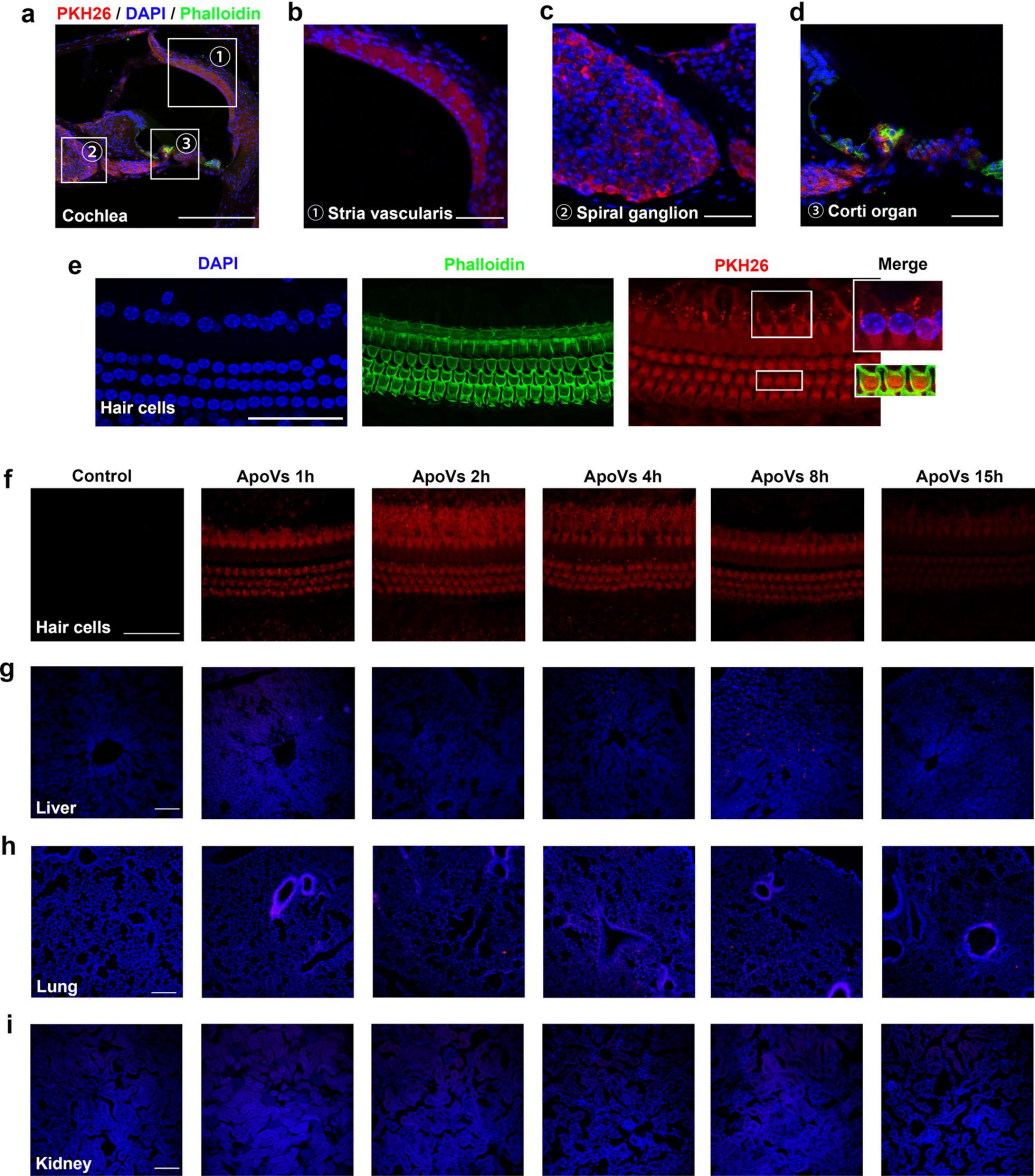
ApoVs distribution features in cochlea, liver, lung and kidney after systemic application. **a, b, c, d, e** representative confocal microscopy images showing 2 h after systemic administration, PKH26-labeled apoVs were distributed in several parts of cochlea (**a**), including stria vascularis (**b**), spiral organ (**c**), corti organ (**d**) and hair cells (**e**). a, scale bar = 200 μm. b, c, d, e, scale bar = 50 μm. Phalloidin staining (green) illustrating the hair cell structure. DAPI staining (blue) for nucleus. ①②③represented the same view of different magnification. **f, g, h, i** representative confocal microscopy images showing distribution of apoVs in hair cells (**f**), liver (**g**), lung (**h**) and kidney (**i**) at several time points after systemic application. f, scale bar = 50 μm. g, h, i scale bar = 200 μm. DAPI staining (blue) for nucleus

Considering the presence of blood–labyrinth barrier, which blocks most systemic drugs out of inner ear and largely reduces their benefits, it is necessary to study whether apoVs can cross blood–labyrinth barrier. We labeled apoVs with PKH26 and injected them into CBA/J mice via tail vein. Surprisingly, 2 h after intravenous injection, PKH26-labeled apoVs were found in several parts of cochlea (Fig. 2a), including stria vascularis (Fig. 2b), spiral ganglion (Fig. 2c) and corti organ (Fig. 2d, e). Then, focusing on hair cells (HCs), we found that PKH26-labeled apoVs were distributed in both inner hair cells (IHCs) and outer hair cells (OHCs) (Fig. 2e). Further, we studied distribution of apoVs at different time points. In HCs, we found that apoVs firstly emerged 1 h after injection, steadily existed in 4 h, and gradually decreased after 8 h (Fig. 2f). Specifically, 2 h after administration, uptake of apoVs raised in HCs especially in IHCs. Between 4 and 8 h after application, apoVs fluorescence dramatically decreased and almost disappeared 15 h after application. These results indicated that 2–4 h after application may be the peak of apoVs uptake in hair cells. We also found that uptake of apoVs showed different patterns in different organs. In liver, a few apoVs were distributed during 1 h and 4 h after application, while 8 h after application, plenty of apoVs were showed and gradually decreased 15 h after application (Fig. 2g). PKH26-labeled apoVs were also seen in lungs 1 h after administration and lasted for 15 h (Fig. 2h). However, few PKH26 fluorescence was showed in kidney from 1 to 15 h after application (Fig. 2i). Understanding the distribution patterns of apoVs can help determine the best administration time and further improve therapeutic effect of apoVs.

### ApoVs restored noise-induced hearing loss by reducing the loss of outer hair cells

**Fig. 3:**
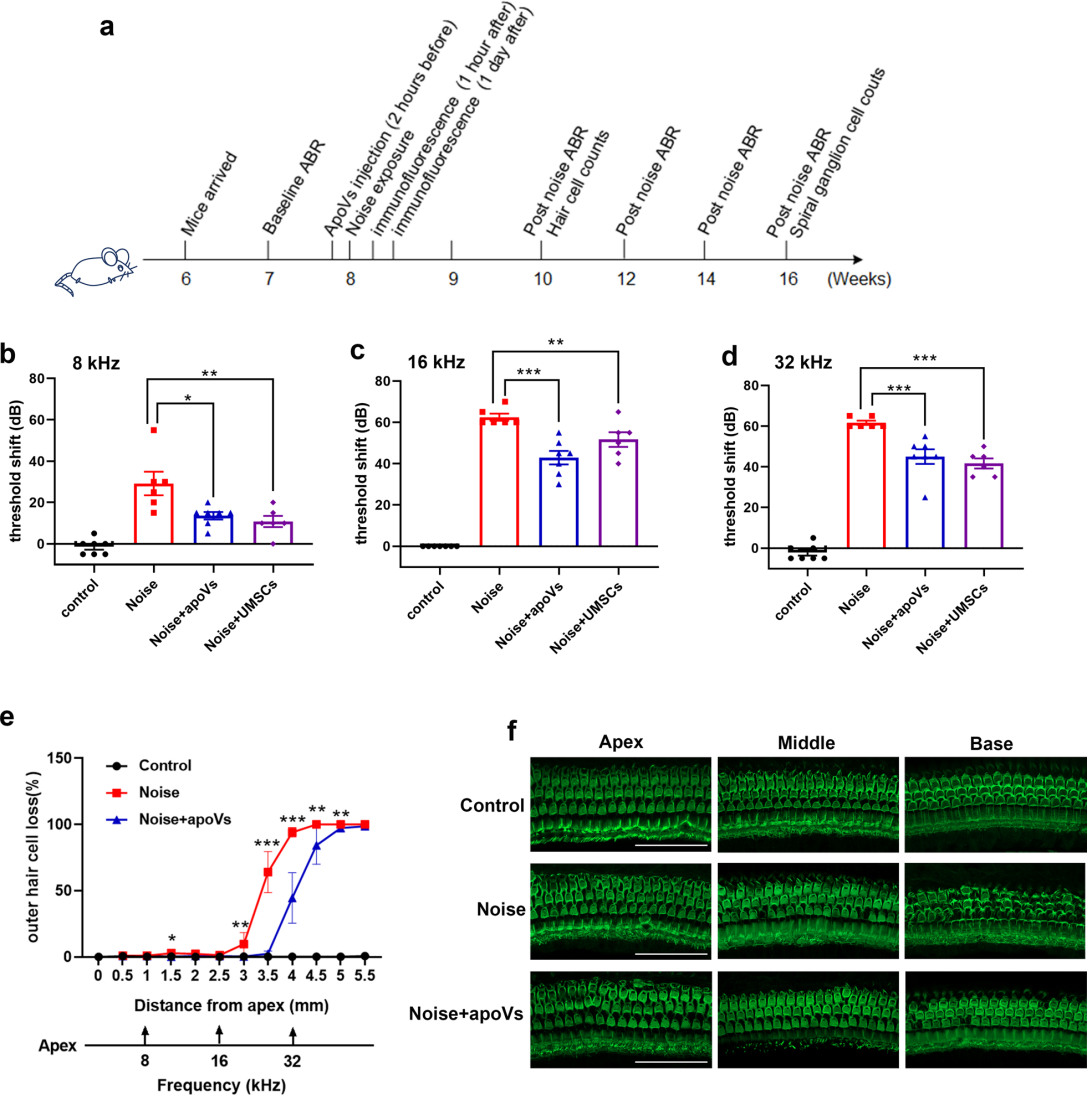
ApoVs restored noise-induce hearing loss and reduced the loss of outer hair cells. **a** schematic diagram indicating the protocol for in vivo experiments. **b, c, d** auditory brainstem response (ABR) measurements showing the auditory threshold shifts of each group at 8 kHz (**b**), 16 kHz (**c**), and 32 kHz (**d**). *N* = 6–7 per group. Data are presented as individual point with means ± SD.** e** a cytocochleogram showing the differences in the number of lost OHCs. *N* = 6–7 per group. * represent the *p* value between Noise and Noise + apoVs group. **f** representative confocal microscopy images of the basilar membrane showing the differences in OHCs in apex, middle and base turn of each group. *N* = 6–7 per group. Scale bar = 50 μm. Data are presented as means ± SD. *p < 0.05, **p < 0.01, ***p < 0.001

To evaluate the therapeutic effect of apoVs therapy in NIHL, we used auditory brainstem response (ABR) test to objectively estimate auditory threshold shifts in CBA/J mice. According to the distribution pattern of apoVs in cochlea, we administered apoVs 2 h before noise exposure, ensuring that apoVs would not dramatically decrease in HCs during the time of a 2-h noise exposure (Fig. 3a). ABR tests showed that 14 days after noise exposure, CBA/J mice presented ABR threshold shifts of approximately 30, 60, and 60 dB at 8, 16, and 32 kHz, respectively. Surprisingly, the administration of apoVs ameliorated ABR threshold shifts by 15,20 and 16 dB at 8, 16 and 32 kHz, respectively, compared with noise group. Also, the administration of UMSCs ameliorated ABR threshold shifts by 18, 10 and 20 dB at 8,16 and 32 kHz, respectively. Notably, there is no significant difference between apoVs and UMSCs treatment (Fig. 3b, c, d). These results demonstrated that noise exposure led to severe hearing impairment, which can be attenuated by pretreatment with apoVs. Considering the main effect of noise exposure is the loss of OHCs, we then performed the counting of OHCs. In line with ABR results, noise exposure caused a loss of OHCs, especially at 3–5.5 mm from the apex along the cochlear duct. Expectedly, apoVs treatment significantly reduced the loss of OHCs at 1.5 mm and 3–5 mm from the apex (Fig. 3e), which was corresponding to the reduction of OHCs loss in middle and base turn of basilar membrane (Fig. 3f). Moreover, to study the long-term effect of apoVs in NIHL, we also performed ABR measurements 4 weeks, 6 weeks and 8 weeks after noise exposure. Results showed that the benefit of apoVs treatment lasted until 8 weeks after noise exposure (Additional file 4: Fig. S3a), suggesting a long-term protection of NIHL. We also found that noise exposure caused a reduction in both hair cells and spiral ganglion cells at 8 weeks after noise exposure, which were partially attenuated by pretreatment with apoVs (Additional file 4: Fig. S3b, c, d). This may because spiral ganglion cells loss was thought to be the secondary damage of hair cell loss [42].

Moreover, to seek for a better benefit of apoVs in NIHL, we study its effect through different administration pathway or at different administration time. However, apoVs treatment after noise exposure did not attenuate NIHL (Additional file 4: Fig. S3e), which may because hair cells experienced irreversible damage immediately after noise exposure and apoVs treatment was not able to regenerate hair cells [43]. What’s more, apoVs treatment through bulla injection showed no protection of NIHL (Additional file 4: Fig. S3f). While the diversity between individuals in each group was large, we considered that the bulla injection is still an invasive operation with side effects on hearing.

### ApoVs administration reduced noise-induced oxidative stress in OHCs

**Fig. 4:**
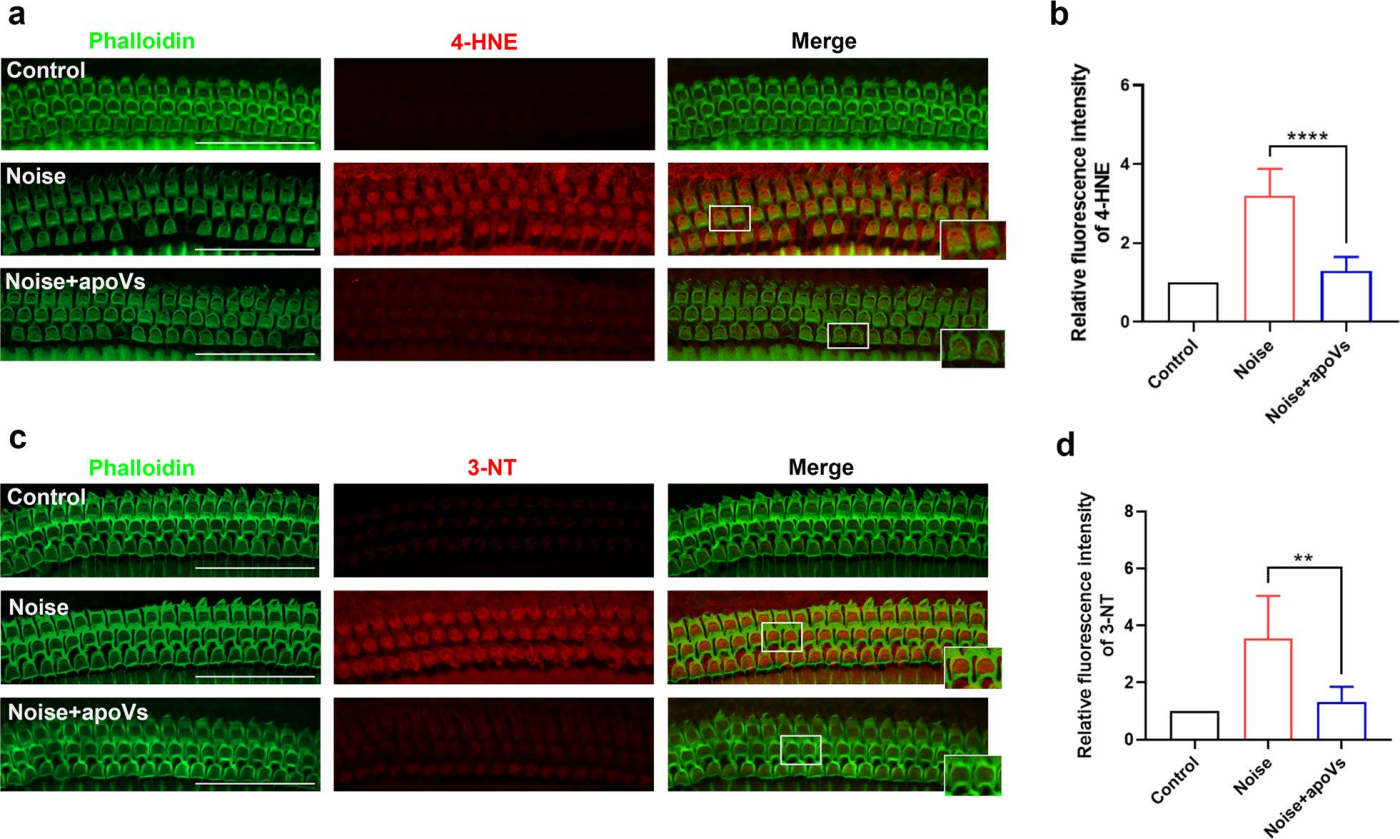
ApoVs treatment reduced noise-induced oxidative stress in OHCs. **a** Representative confocal microscopy images showing the immunofluorescence of 4-HNE in OHCs. Scale bar = 50 μm. Phalloidin staining (green) illustrating the outer hair cells structure. 4-HNE: 4-hydroxynonenal. **b** semi-quantification of fluorescence intensity of 4-HNE in OHCs.* N* = 6 per group. **c** representative confocal microscopy images showing the immunofluorescence of 3-NT in OHCs. Scale bar = 50 μm. Phalloidin staining (green) illustrating the OHCs structure. 3-NT: 3-nitrotyrosine. **d** semi-quantification of fluorescence intensity of 3-NT in OHCs.* N* = 5 per group. Data are presented as means ± SD. *p < 0.05, **p < 0.01, ***p < 0.001

Oxidative stress is an explicit factor for NIHL. For now, there is no evidence on the association between apoVs and oxidative stress. To investigate the possible role of apoVs in resisting oxidative stress, we used 4-hydroxynonenal (4-HNE) and 3-nitrotyrosine (3-NT) as oxidative stress markers and assessed their levels. In our study, the fluorescence intensity of 4-HNE and 3-NT significantly increased in OHCs after noise exposure, suggesting that noise exposure induced serious oxidative damage in OHCs. Surprisingly, administration of apoVs remarkably attenuated the elevation of 4-HNE and 3-NT in OHCs (Fig. 4a, c). Semi-quantification of fluorescence intensity for 4-HNE and 3-NT both confirmed significant increases after noise exposure, which were ameliorated by apoVs administration (Fig. 4b, d). These results indicated that apoVs administration can resist noise-induced oxidative stress in OHCs.

### Endocytosis of apoVs diminished H_2_O_2_-induced HEI-OC1 cell death

**Fig. 5:**
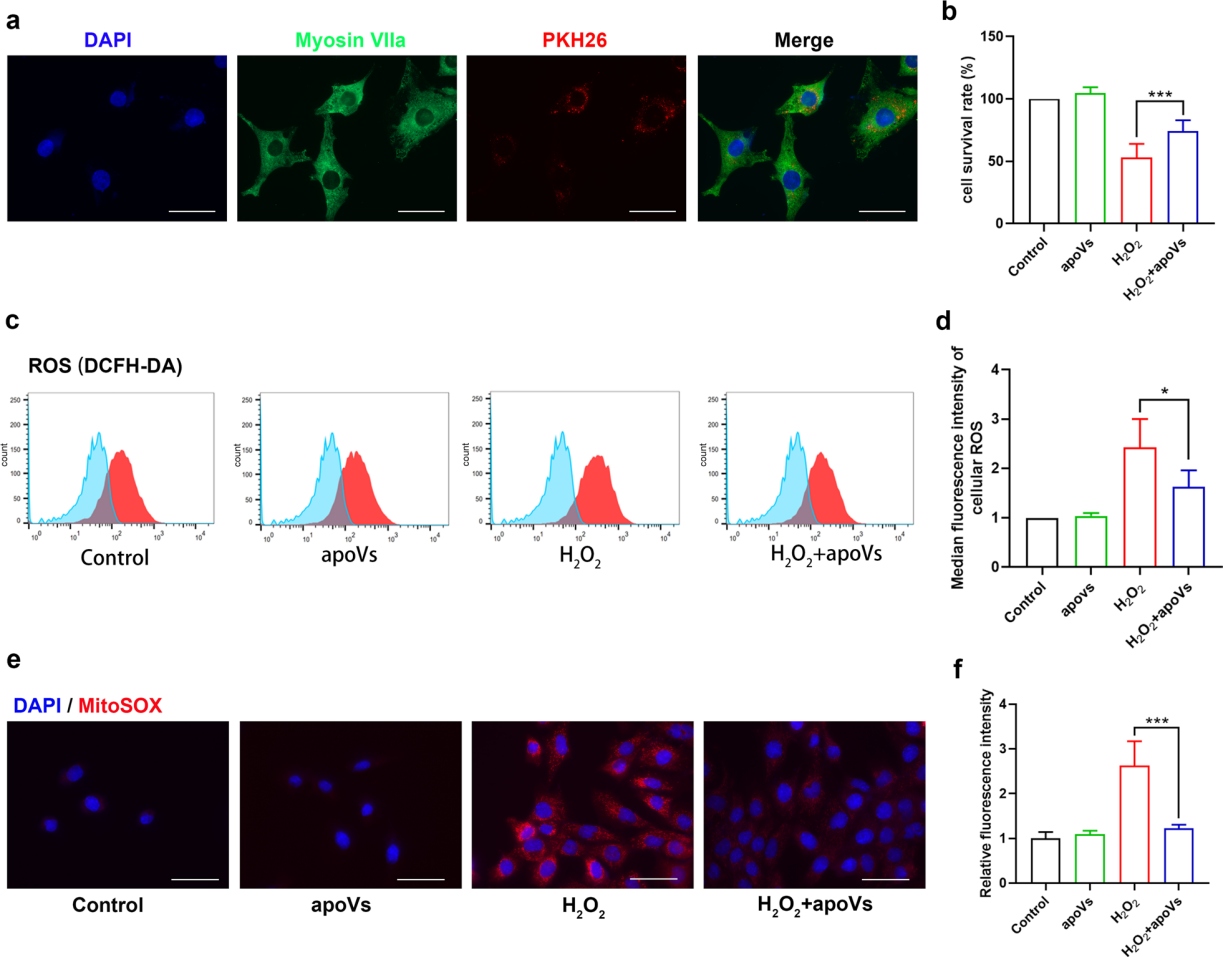
Endocytosis of apoVs diminished H_2_O_2_-induced HEI-OC1 cells death and reduced cellular and mitochondrial ROS. **a** Representative fluorescence microscope images showing endocytosis of PKH26-labeled apoVs in HEI-OC1 cells (green) through co-culture. Scale bar = 50 μm. DAPI staining for nucleus. **b** Cell Counting Kit-8 (CCK8) assay examining the HEI-OC1 cell viability in each group. *N* = 6 per group. **c** flow cytometry assay of DCFH-DA showing the cellular ROS levels in HEI-OC1 cells of each group. **d** quantification of median fluorescence intensity of DCFH-DA confirming the significant difference of cellular ROS levels between each group. *N* = 4 per group. **e** representative fluorescence microscope images of MitoSOX staining (red) showing the mitochondrial ROS levels in HEI-OC1 cells of each group. Scale bar = 50 μm. DAPI staining for nucleus. **f** semi-quantification of MitoSOX fluorescence intensity in HEI-OC1 cells. *N* = 5 per group. Data are presented as means ± SD. *p < 0.05, **p < 0.01, ***p < 0.001

In order to explore the potential mechanisms underlying apoVs-mediated protection against NIHL, we conducted in vitro experiments using HEI-OC1 cell, a commonly used cell line in auditory studies. First, we confirmed that apoVs were endocytosed by HEI-OC1 cells through co-culture (Fig. 5a). Moreover, the endocytosis of apoVs showed a dose-dependent manner and a time-dependent manner. In other words, the higher concentration of apoVs and the longer time of co-coculture, the more apoVs HEI-OC1 cells endocytosed (Additional file 5: Fig. S4). Then, we used H_2_O_2_ treatment (1 mmol/L, 2 h) as a simulation of oxidative stress and analyzed the survival of HEI-OC1 cells through Cell Counting Kit-8 (CCK8) assay. After H_2_O_2_ treatment, only 50% of HEI-OC1 cells survived. Expectedly, pretreatment with apoVs (10 ug/ml, 48 h) reduced HEI-OC1 cell death of approximately 20% (Fig. 5b). These results indicated HEI-OC1 cells can endocytose apoVs and pretreatment with apoVs can diminish H_2_O_2_-induced HEI-OC1 cell death.

### ApoVs reduced cellular and mitochondrial ROS in H_2_O_2_-treated HEI-OC1 cells

Based on our in vivo experiments, we hypothesized that apoVs treatment reduced H_2_O_2_-induced HEI-OC1 cells death by attenuating oxidative stress. To confirm this, we measured the cellular ROS levels, which were detected by DCFH-DA. After quantifying fluorescence intensity by flow cytometry, we found that apoVs treatment can reduce cellular ROS level in H_2_O_2_-treated HEI-OC1 cells by approximately 33% (Fig. 5c, d). It is generally recognized that ROS are produced from oxidative phosphorylation process in mitochondria, which means mitochondria plays important role in producing and cleaning up ROS. Thus, we monitored mitochondria ROS level by MitoSOX staining. From fluorescence microscope, we found that mitochondria ROS level elevated after H_2_O_2_ exposure, which was attenuated by apoVs treatment (Fig. 5e). Semi-quantification of fluorescence intensity also supported our results (Fig. 5f). These suggested that apoVs attenuated oxidative stress and cellular ROS elevation in H_2_O_2_-treated HEI-OC1 cells by reducing mitochondria ROS.

### ApoVs upregulated SOD2 and promoted FOXO3a nuclear translocation

**Fig. 6:**
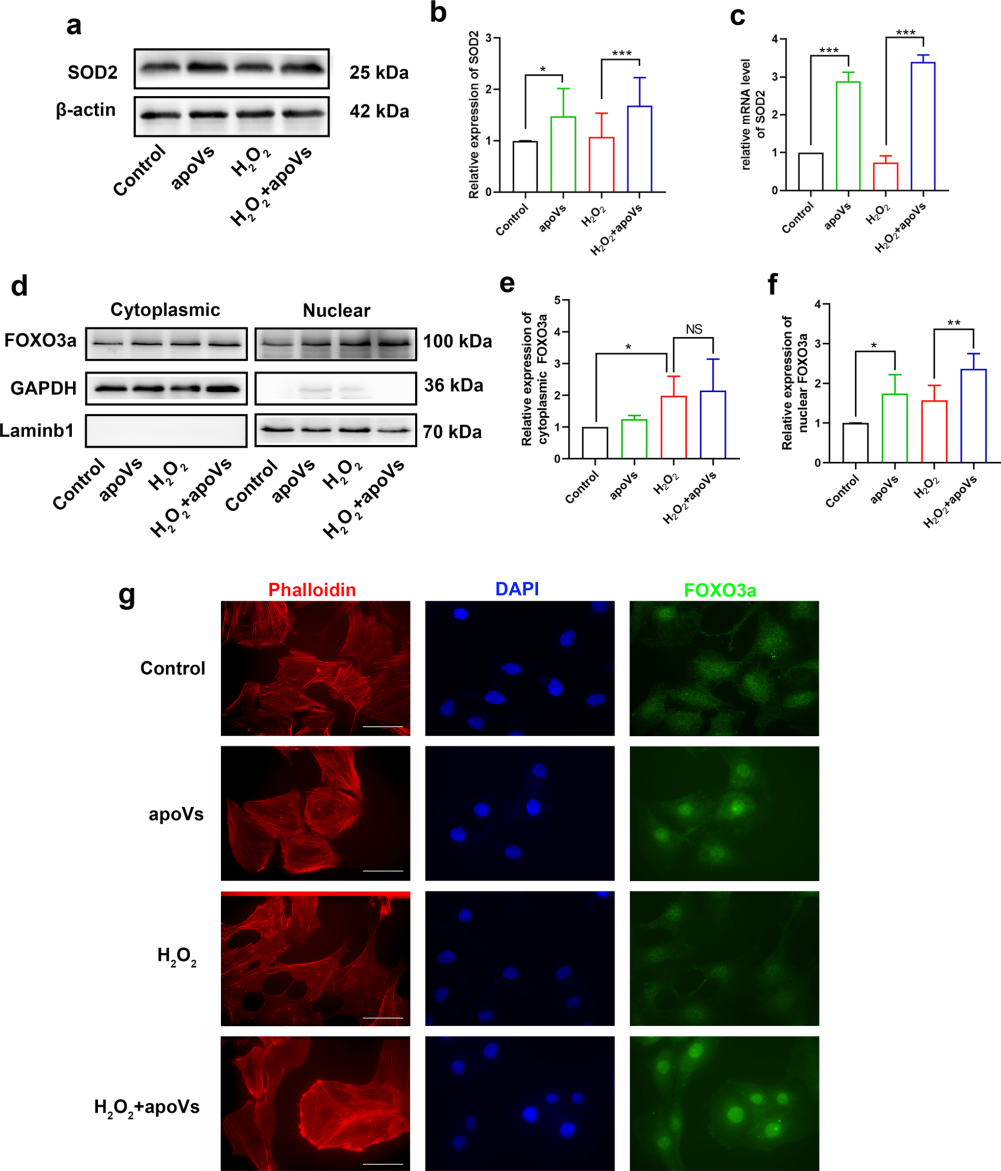
ApoVs upregulated SOD2 and promoted FOXO3a nuclear translocation. **a, b** Western-blotting analyzed the SOD2 expression in HEI-OC1 cells of each group (**a**), and statistical analysis (**b**). *N* = 8 per group. **c** RT-PCR analysis indicating the transcriptional levels of SOD2. *N* = 4 per group. **d, e, f** western-blotting of cytoplasmic and nuclear extracts from HEI-OC1 cells showing the expression of FOXO3a, GAPDH and Laminb1 (**d**), and statistical analysis (**e, f**). The expression of cytoplasmic (**e**) and nuclear (**f**) FOXO3a were relative to GAPDH and Laminb1 respectively. *N* = 4 per group. **g** representative fluorescence microscope images showing the location of FOXO3a protein in HEI-OC1 cells of each group. Scale bar = 50 μm. Phalloidin staining (red) illustrating the HEI-OC1 cells structure. DAPI staining (blue) for nucleus. Data are presented as means ± SD. *p < 0.05, **p < 0.01, ***p < 0.001, NS, no significant difference

Now that apoVs can reduce mitochondrial ROS level, we assumed that apoVs upregulated enzymes in mitochondria which related to the elimination of ROS. Thus, we recognized SOD2, an antioxidant enzyme in mitochondria, which can catalyze the transformation from free radicals superoxide O_2_^·−^ to O_2_ and H_2_O_2_. Expectedly, western blotting showed that SOD2 expression level significantly increased in HEI-OC1 cells after apoVs treatment (Fig. 6a, b). Also, RT-PCR results showed the mRNA level of SOD2 elevated in apoVs and H_2_O_2_ co-treatment group compared with H_2_O_2_ group (Fig. 6c), indicating that SOD2 gene transcription was promoted by apoVs treatment. As a result, we looked through transcription factors which can regulate transcription of SOD2 gene according to previous studies, in which we found out FOXO3a (Forkhead box O3) [44]. The location of FOXO3a in cells depends on its activation state. FOXO3a locates in cytoplasm in inactivated state while translocating into nucleus after activation. Thus, to study the distribution of FOXO3a after apoVs treatment, we isolated cytoplasm and nuclear extracts from HEI-OC1 cells and detected FOXO3a protein levels in cytoplasm and nucleus respectively. Western blotting showed that H_2_O_2_ treatment can improved cytoplasmic FOXO3a levels while apoVs treatment showed no significantly effect on cytoplasmic FOXO3a levels (Fig. 6d, e). As for changes of nucleus FOXO3a protein levels, apoVs treatment significantly increased nucleus FOXO3a level compared with control group or H_2_O_2_-treated group (Fig. 6d, f). Immunofluorescence also proved that FOXO3a protein accumulated into nucleus after apoVs treatment (Fig. 6g). These results indicated that apoVs treatment activated SOD2 transcription by promoting FOXO3a nuclear translocation.

### STAT3 may be responsible for activation of FOXO3a-SOD2 pathway

**Fig. 7:**
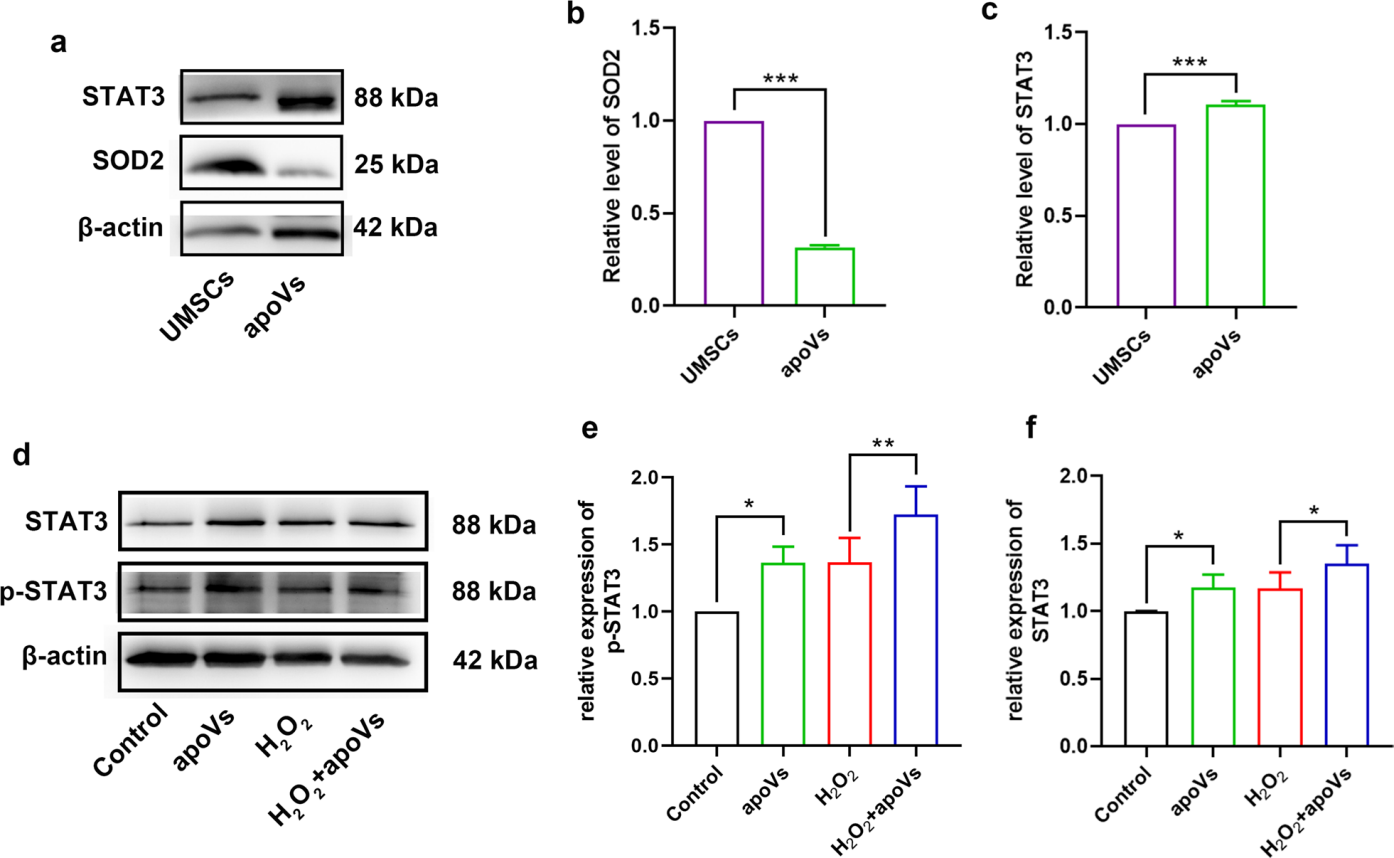
STAT3 in apoVs may responsible for activating FOXO3a-SOD2 pathway. **a, b, c** western blotting examining the STAT3, SOD2, β-actin protein levels in UMSCs and apoVs (**a**), and statistical analysis (**b, c**). The level of SOD2 (**b**) and STAT3 (**c**) were relative to β-actin. *N* = 3 per group. **d, e** western blotting showing the STAT3, p-STAT3, β-actin levels in HEI-OC1 cells of each group (**d**), and statistical analysis (**e, f**). *N* = 4 per group. Data are presented as means ± SD. *p < 0.05, **p < 0.01, ***p < 0.001

Now that apoVs can activate FOXO3a-SOD2 pathway, we assumed that some functional proteins in apoVs are responsible for its activation. Although we recognized SOD2 from proteomics results of apoVs (Table 2), western blotting confirmed that the level of SOD2 in apoVs were significantly lower than MSCs (Fig. 7a, b). From proteomics results, we also recognized STAT3(signal transduction and activators of transcription 3) (Table 2), a transcription factor responding to several cytokines or growth factors. Moreover, STAT3 was enriched in apoVs confirmed by western blot (Fig. 7a, c). Plenty of evidence showed that it is related to FOXO3a nuclear translocation [45, 46]. Previous study showed that phosphorylated STAT3 interacted with FOXO3a in cytoplasm and promoted its nuclear translocation in CD4^+^ T cells [47]. However, its association with FOXO3a-SOD2 pathway in hair cells haven’t been studied yet. Western blotting showed the level of STAT3 and p-STAT3 increased after apoVs treatment (Fig. 7d). Statistical analysis also proved a significantly increase of STAT3 and p-STAT3 in apoVs-treated group compared to control and H_2_O_2_-treated group (Fig. 7e, f). These suggested that STAT3 in apoVs may be the functional protein activating FOXO3a nuclear translocation and SOD2 upregulation.

**Table 2:** Characterized SOD2 and STAT3 and related records from proteomics analysis

Protein ID	Protein names	Gene names	Number of proteins	Unique peptide	Sequence coverage D540.raw (%)	Score
P04179	Superoxide dismutase [Mn], mitochondria	SOD2	20	4	20.7	6.0264
P40763	Signal transducer and activator of transcription 3	STAT3	18	1	2.8	1.2728

## Discussion

Noise-induced hearing loss (NIHL) has been a universal problem for clinical systems. Although there is rising population suffering NIHL, few drugs can be used to treat it [48]. Thus, MSC transplantation especially extracellular vesicles transplantation is becoming an attractive therapy for NIHL. Recently, the role of apoptosis in MSCs therapy was put forward and MSC-derived apoVs have been proved to be a potential therapy for several diseases, indicating a new cell-free therapy for NIHL. As a result, we studied the effect of MSC-derived apoVs on NIHL and the potential mechanism against oxidative stress.

In otology, because of the existence of blood-labyrinth barrier, few drugs can go into inner ear through systemic administration, which strongly limits their benefits [49]. Methods for inner ear delivery included intra-tympanic injection, intra-labyrinthine injection and artificial nanocarriers [50]. However, intra-tympanic and intra-labyrinthine injection are invasive operations. Artificial nanocarriers are far from clinical use because of its unknown bioavailability [51]. Surprisingly, we found that apoVs enriched in cochlea especially stria vascularis, spiral ganglion cells and hair cells after systematic administration. Besides their smaller diameter, the mechanism behind may relate to a new-found target for blood-labyrinth barrier, LRP1 (low-density lipoprotein receptor-related protein 1) [52], which was also identified in apoVs according to our proteomics analysis (Additional file 1: Table S1). What’s more, apoVs showed more advantages in inner ear delivery. Compared to nanoparticle delivery, these natural, cell-derived extracellular vesicles exhibited intrinsic payload and better bioavailability [53]. Researches have proved the great value of extracellular vesicles in inner ear delivery, for example, it can be used for gene transfer via adeno-associated viruses (AAV) [54]. From our study, MSCs-derived apoVs contained several proteins and naturally showed great therapeutic effect on NIHL. It is totally reasonable to upregulate specific functional proteins in apoVs in order to achieve a better benefit. As a result, apoVs may be a prospective drug delivery system for inner ear.

Previous studies have proved that MSCs transplantation and MSC-derived exosome therapy are beneficial to various sensorineural hearing loss too, but their distinction between apoVs therapy has not been studied yet. Our study found that apoVs derived from MSCs performed the same benefit as MSCs in preventing NIHL, which supported the theory that after transplantation, MSCs went through spontaneously apoptosis and released apoVs that truly exert therapeutic function [55]. As apoVs therapy possessed advantages including lower immunogenicity, easier storage and less coagulation risk, it may be a more direct and promising therapy than MSC transplantation. As for the difference between MSCs-derived exosomes and apoVs, recent study has disclosed their different proteomics pattern. Researchers found that compared to exosomes, various functional proteins were enriched in apoVs, including STAT3. These upregulated proteins in apoVs were mainly associated with “cell growth and death,” “signal transduction,” “translation,” “cancers,” “global and overview maps,” and “immune system” [56]. Besides, apoVs extraction required easier condition, shorter time with larger production. Extraction of apoVs required a maximum centrifugal force of only 16,000 g for 30 min to which a high-speed centrifuge was totally sufficient while extraction of exosomes required ultracentrifuge and a maximum centrifugal force of 100,000 g for 70 min [57]. These advantages suggested that apoVs may be a more suitable cell-free therapy for massive clinical use.

Oxidative stress is an explicit factor for NIHL. After noise exposure, cochlea experienced oxidative stress, which finally led to obstruction of stria vascularis and death of hair cells [36, 58]. SOD2, an oxidoreductase located in mitochondria, have been considered as an initial enzyme for cleaning up ROS by catalyzing O_2_^·−^ [59]. Previous studies have proved that upregulation of SOD2 can resist oxidative stress and attenuate hearing loss [60, 61]. In our study, we found that apoVs not only carried SOD2, but also promoted cellular SOD2 transcription to resist oxidative stress caused by noise exposure. Interestingly, recent studies stated that the primary role of SOD2 in resisting oxidative stress was not an oxidoreductase but a signal transductor. They suggested that SOD2 may be important in activating oxidant signaling pathways because SOD2 catalyzes O_2_^·−^ into H_2_O_2_, which is a freely diffusible oxidant compared to O_2_^·−^. H_2_O_2_ diffused to cytoplasm and regulated functional proteins by oxidation of their cysteine residues [62], which can activate biological pathways such as cell proliferation [63]. Then, the cysteine residues oxidation of functional proteins can recover through disulfide reductases, thioredoxin and glutaredoxin [64]. These oxidation proteins served as a reversible signal for activate signaling pathways which promoted adaptation to elevated superoxide [65] while H_2_O_2_ itself was further eliminated by cellular antioxidative proteins such as peroxiredoxins, glutathione peroxidase, and catalase. These studies emphasized a unique role of SOD2 in resisting oxidative stress.

FOXO3a-SOD2 pathway has been studied in other organs or tissues and proved to effectively eliminate mitochondrial or cellular ROS [44, 66, 67]. In sensorineural hearing loss, researchers found that FOXO3a-SOD2 pathway was inhibited along with an increasing ROS level [68, 69]. What’s more, our previous study found that upregulation of FOXO3a can protect against cisplatin-induced hearing loss [70], suggesting the significance of FOXO3a in resisting hearing loss. In this study, we found that apoVs treatment successfully upregulated FOXO3a-SOD2 pathway by promoting FOXO3a nuclear translocation. Also, we recognized that STAT3, a member of signal transduction and activators of transcription family, was enriched in apoVs. Previous studies showed that STAT3 can promote FOXO3a nuclear translocation in CD4^+^T cells [47], but their relationship in hair cells haven’t been studied yet. In our study, after apoVs treatment, STAT3 upregulated in HEI-OC1 cells, along with the upregulation of its activation subtype p-STAT3 (Tyr705), suggesting that STAT3 in apoVs was important in resisting noise-induced oxidative stress.

However, because of the complicated components in apoVs, whether the STAT3-FOXO3a-SOD2 pathway is the only effective pathway against NIHL needs to be further investigated. What’s more, there were some limitations in our animal NIHL model. While noise exposure was usually chronic damage in reality life, our NIHL model was an acute damage. But we proved that apoVs treatment can upregulate SOD2 and ameliorate oxidative stress, which were proved to be risk factors of NIHL from clinical evidence [27, 28], suggesting that apoVs treatment may be effective in clinical use.

## Conclusions

In conclusion, our findings demonstrate that MSC-derived apoVs are able to cross blood–labyrinth barrier and resist oxidative damage caused by noise exposure. Further, apoVs promotes FOXO3a nuclear translocation and activates SOD2 expression, which STAT3 in apoVs may be responsible for. These findings propose a promising way for inner ear delivery and a prospective cell-free therapy for noise-induced hearing loss.

## Supplementary Information

**Additional file 1**. Table S1 List of all the proteins identified from proteomic analysis.

**Additional file 2**. Fig. S1 Characterization of MSCs and MSC-derived apoVs. a analysis of MSC surface markers by flow cytometry. Numbers on top indicated the percentage of positive cells. b crystal violet staining and representative images showing the colony formation ability of MSCs. Scale bar = 500 μm. c Oil red O (ORO) staining and alizarin red S staining demonstrating osteogenic differentiation and adipogenic differentiation in MSCs. Scale bar = 200 μm. d representative bright field images of MSCs and staurosporine (STS)-induced apoptotic MSCs. Scale bar = 500 μm. e a sketch map about the protocol for extraction of MSC-derived apoVs. STS, staurosporine. f representative transmission electron microscope image of apoVs morphology. Scale bar = 500 nm. g nanoparticle tracking analysis (NTA) of apoVs diameter distribution. Numbers indicated the particle diameter of each peak. h western blotting analysis of MSCs and apoVs revealing caspase-3 and cleaved caspase-3.

**Additional file 3**. Fig. S2 GO enrichment analysis of identified proteins in apoVs. The top twenty enriched terms of categories ‘Biological Process’ (a), ‘Molecular Function’(b), ‘Cellular Component’(c) were respectively presented as bubble charts. The Y-axis represents GO terms and the X-axis represents rich factor. The color of the bubble represents enrichment significance and the size of the bubble represents number of identified proteins.

**Additional file 4**. Fig. S3 Effect of apoVs treatment to noise-induced hearing loss in different conditions. a auditory brainstem response (ABR) measurements showing the auditory threshold shifts of each group at 2 weeks, 4 weeks, 6 weeks and 8 weeks after noise exposure. *N* = 4-5 per group. * represent the *p* value between Noise and Noise+apoVs group at each time point. b statistical analysis showing the difference in hair cell loss. c, d representative H&E staining images from microscope showing the spiral ganglion of Control, Noise and Noise+apoVs group (c), and the difference in the number of spiral ganglion cells (d). Scale bar =1 mm. *N* = 5-6 per group. e ABR measurements showing the auditory threshold shifts of each group. *N* = 3 per group. f ABR measurements showing the auditory threshold shifts of each group. *N* =3-4 per group. Data are presented as means±SD. *p < 0.05, **p < 0.01, ***p < 0.001, NS, no significant difference.

**Additional file 5**. Fig. S4 Uptake of apoVs in HEI-OC1 cells in different conditions. Representative images showing uptake of PKH26-labeled apoVs (5 ug/ul) in HEI-OC1 cells 2, 4, 6, 8, 10, 12, 15 hours after co-culture (a) and uptake of PKH26-labeled apoVs (5, 10, 20, 40, 60, 80, 100 ug/ul) 3 hours after co-culture (b). Scale bar = 50 μm. Myosin-VIIa (green) staining for HEI-OC1 cells. DAPI (blue) staining for nucleus.

**Additional file 6**. The ARRIVE guidelines 2.0: author checklist.

**Additional file 7**. A document of original western blot images.

## Data Availability

All data generated during this study are included in this article and supplementary files. Original blots were included in Additional file 7. The mass spectrometry proteomics data have been deposited to the ProteomeXchange Consortium (http://proteomecentral.proteomexchange.org) via the iProX partner repository [71, 72] with the dataset identifier PXD040989.

## Abbreviations

MSC: Mesenchymal stem cell
NTA: Nanoparticle tracking analysis
NIHL: Noise-induced hearing loss
FDR: False discovery rate
apoVs: Apoptotic vesicles
GO: Gene Ontology
ABR: Auditory brainstem response
KEGG: Kyoto Encyclopedia of Genes and Genomes
RT-PCR: Reverse transcription-polymerase chain reaction
EDTA: Ethylenediaminetetraacetic acid
FOXO3a: Forkhead box o3
TDT: Tucker Davis Technology
SOD2: Mitochondrial superoxide dismutase 2
SPL: Sound pressure level
STAT3: Signal transduction and activators of transcription 3
4-HNE: 4-Hydroxynonenal
YLDs: Years lived with disability
3-NT: 3-Nitrotyrosine
HC: Hair cell
H&E: Hematoxylin–eosin
ROS: Reactive oxygen species
DMEM: Dulbecco's modified Eagle medium
H_2_O_2_: Hydrogen peroxide
CCK8: Cell counting kit-8
SPF: Specific pathogen-free
HBSS: Hank's Balanced Salt Solution
UMSC: Umbilical mesenchymal stem cells
PVDF: Polyvinylidene fluoride
α-MEM: Alpha Minimum Essential Medium
BSA: Bovine serum albumin
FBS: Fetal bovine serum
TBS: Triethanolamine-buffered saline solution
PBS: Phosphate-buffered saline
TBST: Triethanolamine-buffered saline solution with Tween-20
PFA: Paraformaldehyde
IHC: Inner hair cell
ORO: Oil red O
OHC: Outer hair cell
STS: Staurosporine
LRP1: Low-density lipoprotein receptor-related protein 1
TEM: Transmission electron microscope
AAV: Adeno-associated viruses
