# Supplementary figures and images for: Apoptotic vesicles resist oxidative damage in noise-induced hearing loss through activation of FOXO3a-SOD2 pathway
Source: Stem Cell Res Ther. 2023 Apr 15;14:88. doi: 10.1186/s13287-023-03314-7 (PMC10105953; doi:10.1186/s13287-023-03314-7)

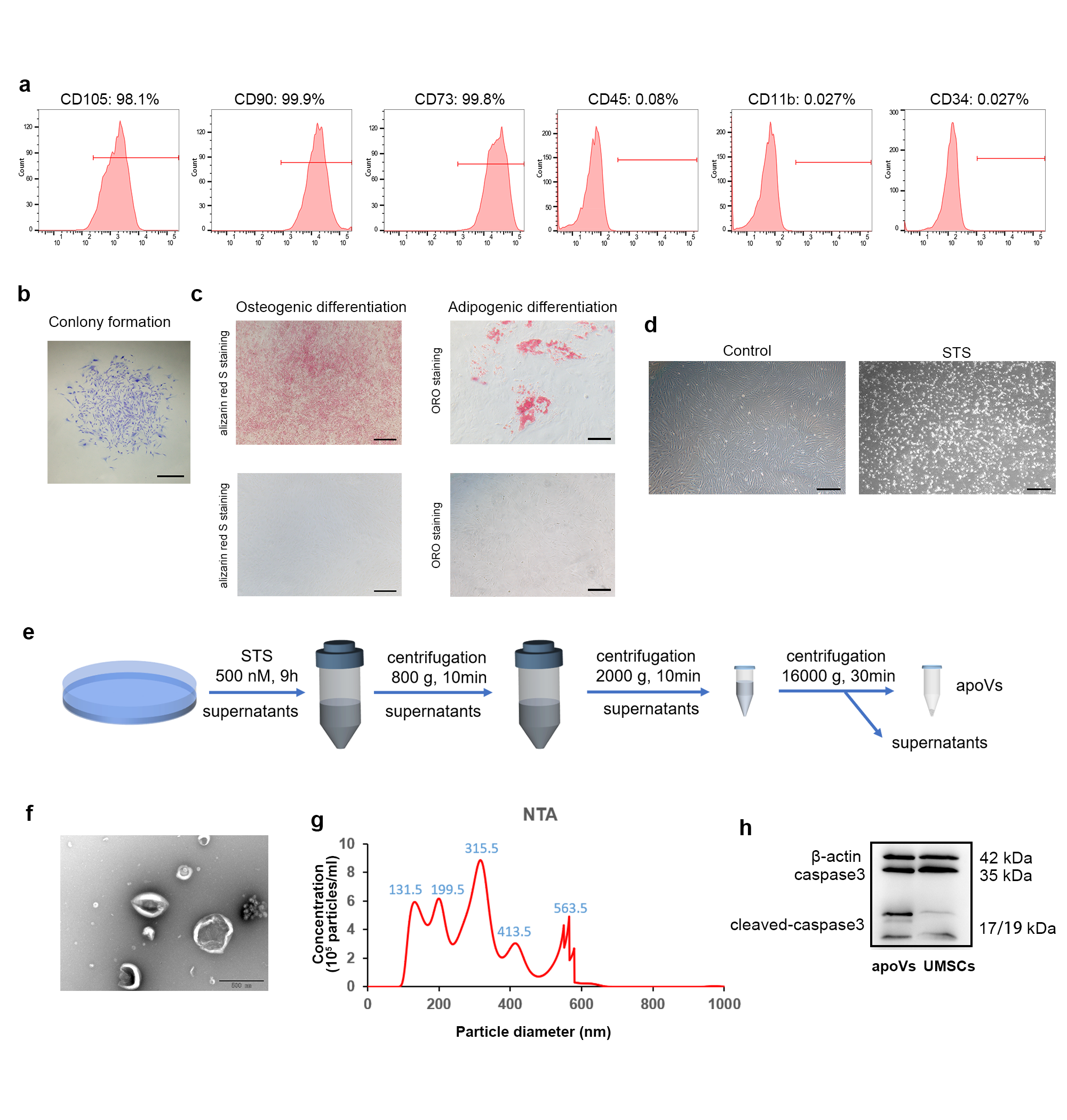

Supplement: Supplementary file 2 — Additional file 2. Fig. S1 Characterization of MSCs and MSC-derived apoVs. a analysis of MSC surface markers by flow cytometry. Numbers on top indicated the percentage of positive cells. b crystal violet staining and representative images showing the colony formation ability of MSCs. Scale bar = 500 μm. c Oil red O (ORO) staining and alizarin red S staining demonstrating osteogenic differentiation and adipogenic differentiation in MSCs. Scale bar = 200 μm. d representative bright field images of MSCs and staurosporine (STS)-induced apoptotic MSCs. Scale bar = 500 μm. e a sketch map about the protocol for extraction of MSC-derived apoVs. STS, staurosporine. f representative transmission electron microscope image of apoVs morphology. Scale bar = 500 nm. g nanoparticle tracking analysis (NTA) of apoVs diameter distribution. Numbers indicated the particle diameter of each peak. h western blotting analysis of MSCs and apoVs revealing caspase-3 and cleaved caspase-3. [file 13287_2023_3314_MOESM2_ESM.tif]

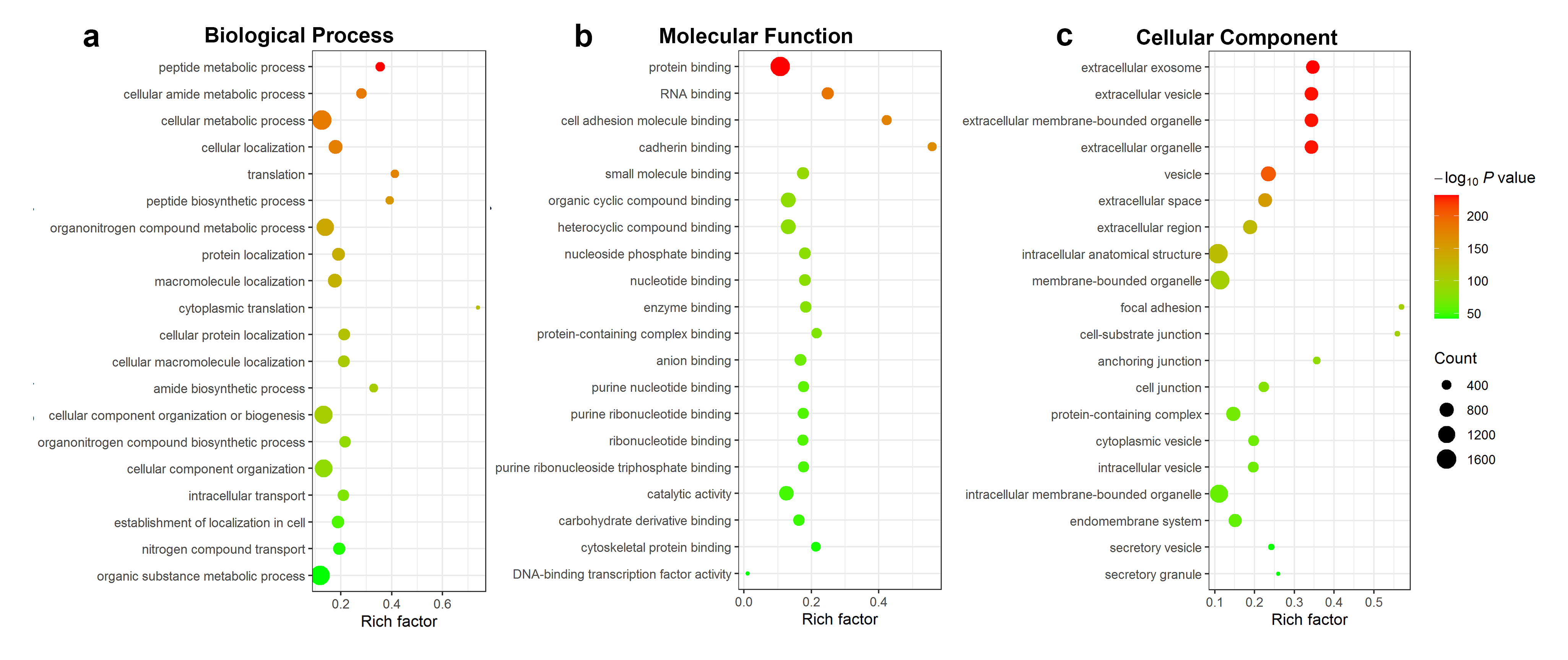

Supplement: Supplementary file 3 — Additional file 3. Fig. S2 GO enrichment analysis of identified proteins in apoVs. The top twenty enriched terms of categories ‘Biological Process’ (a), ‘Molecular Function’(b), ‘Cellular Component’(c) were respectively presented as bubble charts. The Y-axis represents GO terms and the X-axis represents rich factor. The color of the bubble represents enrichment significance and the size of the bubble represents number of identified proteins. [file 13287_2023_3314_MOESM3_ESM.tif]

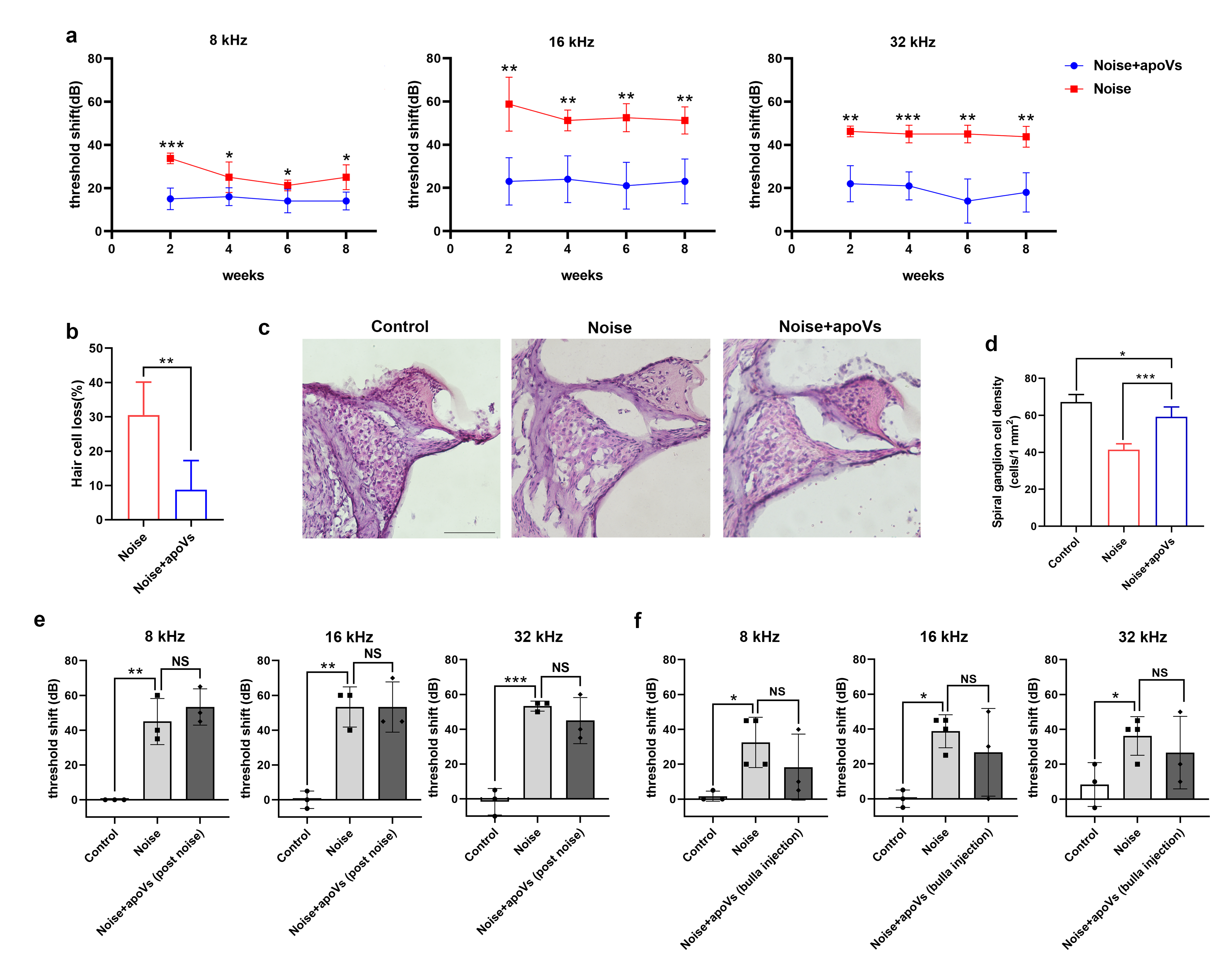

Supplement: Supplementary file 4 — Additional file 4. Fig. S3 Effect of apoVs treatment to noise-induced hearing loss in different conditions. a auditory brainstem response (ABR) measurements showing the auditory threshold shifts of each group at 2 weeks, 4 weeks, 6 weeks and 8 weeks after noise exposure. N = 4-5 per group. * represent the p value between Noise and Noise+apoVs group at each time point. b statistical analysis showing the difference in hair cell loss. c, d representative H&E staining images from microscope showing the spiral ganglion of Control, Noise and Noise+apoVs group (c), and the difference in the number of spiral ganglion cells (d). Scale bar =1 mm. N = 5-6 per group. e ABR measurements showing the auditory threshold shifts of each group. N = 3 per group. f ABR measurements showing the auditory threshold shifts of each group. N =3-4 per group. Data are presented as means±SD. *p < 0.05, **p < 0.01, ***p < 0.001, NS, no significant difference. [file 13287_2023_3314_MOESM4_ESM.tif]

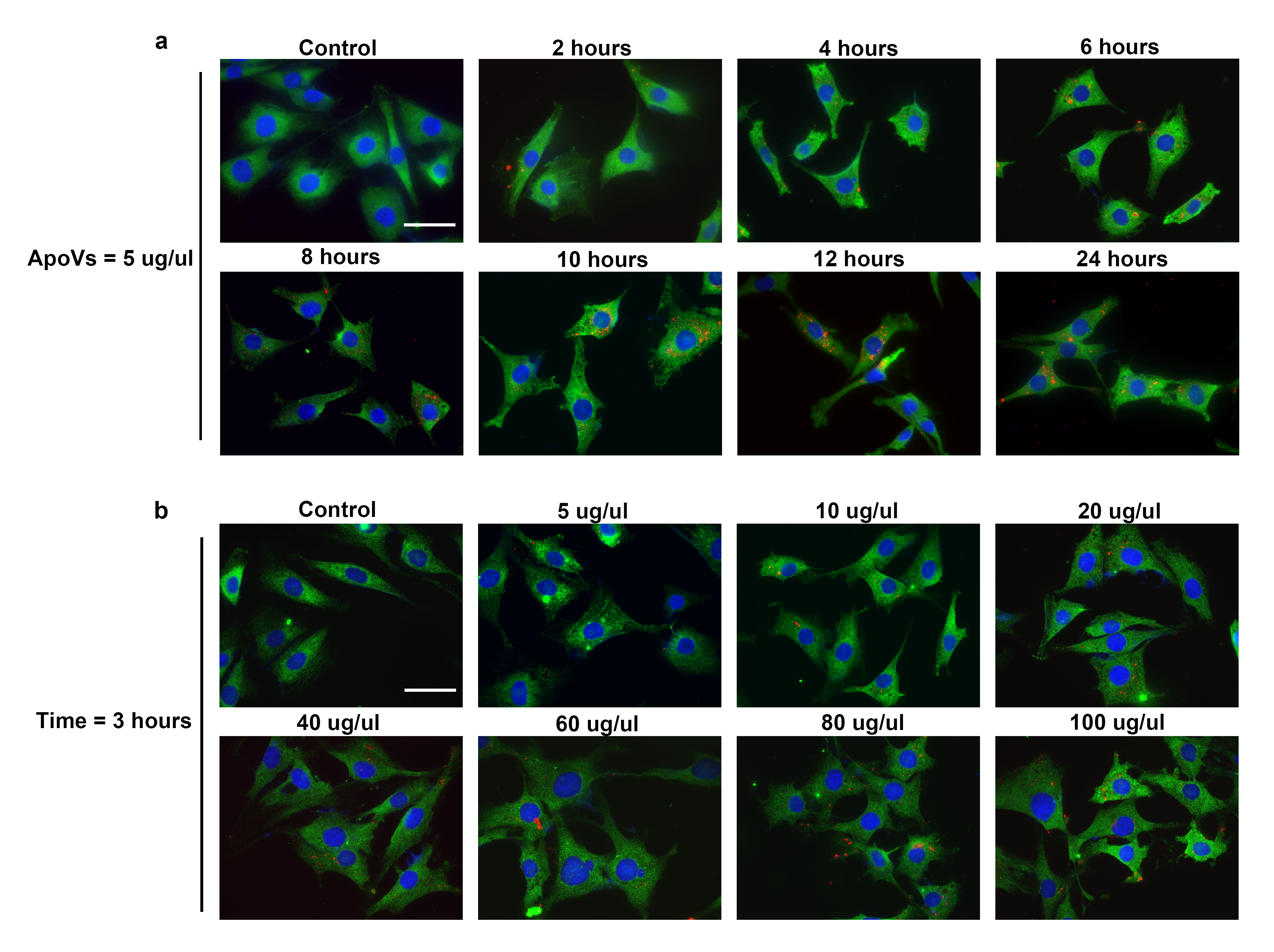

Supplement: Supplementary file 5 — Additional file 5. Fig. S4 Uptake of apoVs in HEI-OC1 cells in different conditions. Representative images showing uptake of PKH26-labeled apoVs (5 ug/ul) in HEI-OC1 cells 2, 4, 6, 8, 10, 12, 15 hours after co-culture (a) and uptake of PKH26-labeled apoVs (5, 10, 20, 40, 60, 80, 100 ug/ul) 3 hours after co-culture (b). Scale bar = 50 μm. Myosin-VIIa (green) staining for HEI-OC1 cells. DAPI (blue) staining for nucleus. [file 13287_2023_3314_MOESM5_ESM.tif]
