# Supplementary material for: Apoptotic vesicles resist oxidative damage in noise-induced hearing loss through activation of FOXO3a-SOD2 pathway
Source: Stem Cell Res Ther. 2023 Apr 15;14:88. doi: 10.1186/s13287-023-03314-7 (PMC10105953; doi:10.1186/s13287-023-03314-7)
Supplement: Supplementary file 7 — Additional file 7. A document of original western blot images. [file 13287_2023_3314_MOESM7_ESM.docx]

|  | 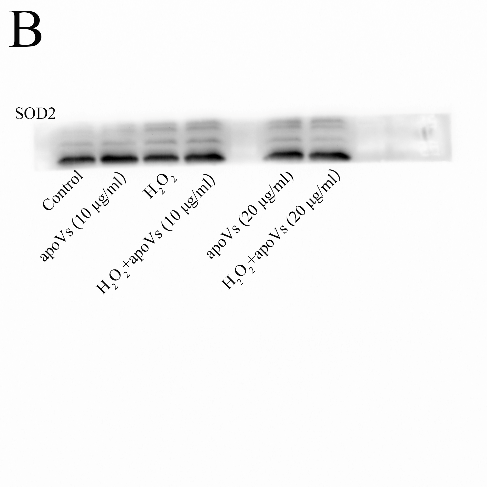 |
| --- | --- |
|  | 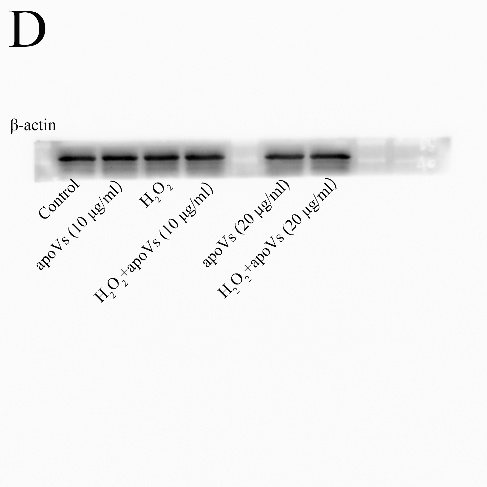 |

Figure 6a. Western-blotting image of SOD2 and β-actin in HEI-OC1 cells of each group. A-B light field image(A) and exposure image(B) of SOD2. C-D light field image(C) and exposure image(D) of β-actin.

|  | 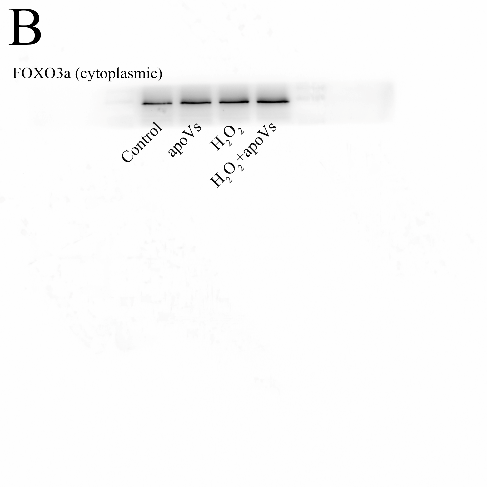 |
| --- | --- |
|  | 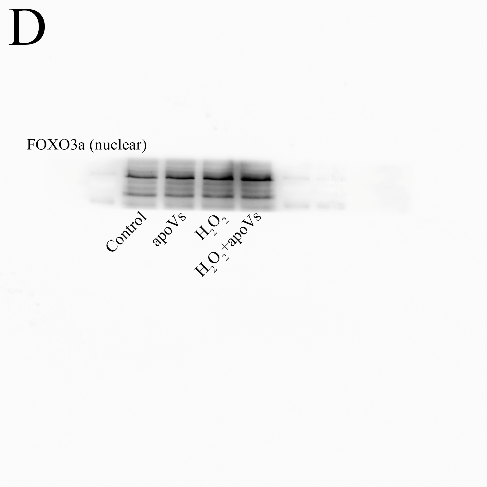 |
|  | 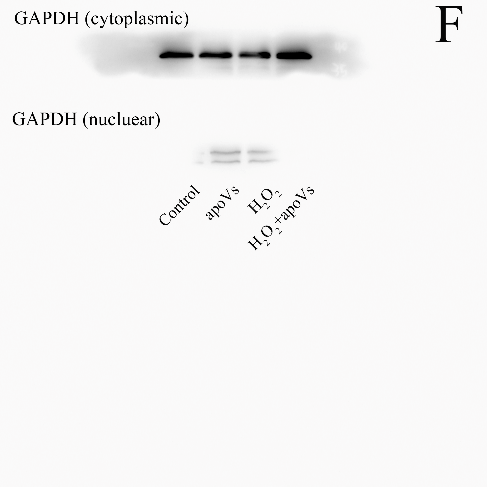 |
|  | 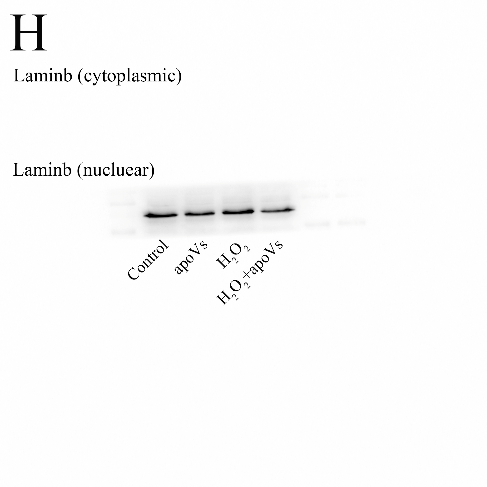 |

Figure 6d. Western-blotting of cytoplasmic and nuclear extracts from HEI-OC1 cells showing the expression of FOXO3a, GAPDH and Laminb1. A-B light field image(A) and exposure image(B) of FOXO3a in cytoplasmic extracts. C-D light field image(C) and exposure image(D) of FOXO3a in nuclear extracts. E-F light field image(E) and exposure image(F) of GAPDH in cytoplasmic and nuclear extracts. G-H light field image(G) and exposure image(H) of Laminb1 in cytoplasmic and nuclear extracts.

|  | 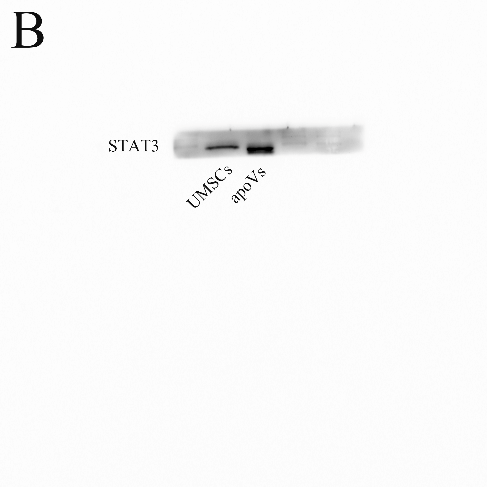 |
| --- | --- |
|  | 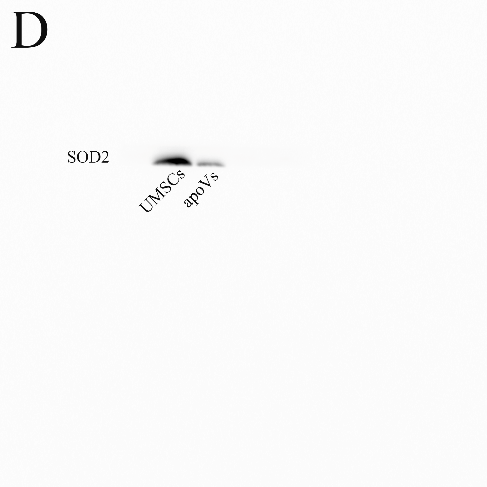 |
|  | 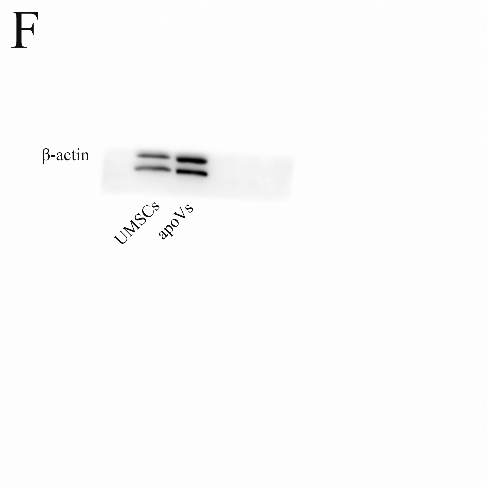 |

Figure 7a. Western blot image of STAT3, SOD2 and β-actin in UMSCs and apoVs. A-B light field image(A) and exposure image(B) of STAT3. C-D light field image(C) and exposure image(D) of SOD2. E-F light field image(E) and exposure image(F) of β-actin.

|  | 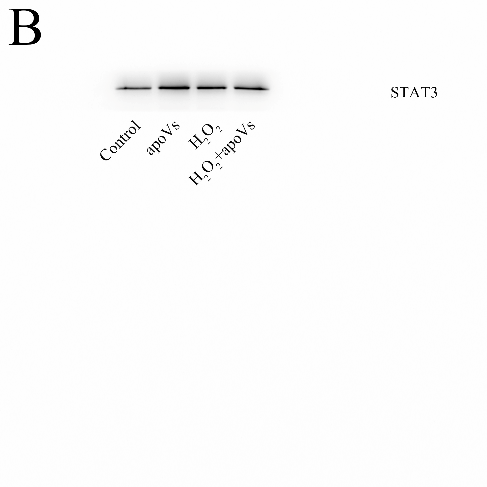 |
| --- | --- |
|  | 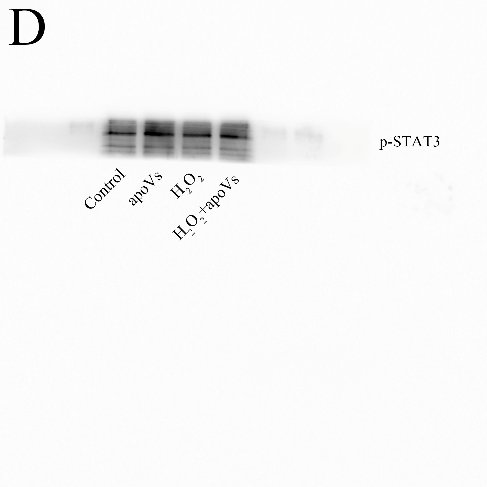 |
|  | 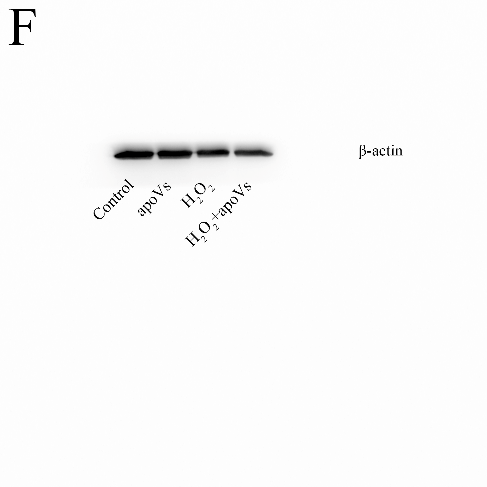 |

Figure 7d. Western blot image of STAT3, p-STAT3, β-actin in HEI-OC1 cells of each group. A-B light field image(A) and exposure image(B) of STAT3. C-D light field image(C) and exposure image(C) of p-STAT3. E-F light field image(E) and exposure image(F) of β-actin.

|  | 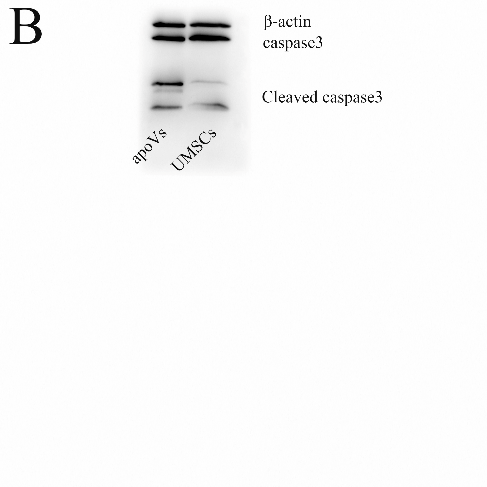 |
| --- | --- |

Figure S1h. Western blot image of caspase-3, cleaved caspase-3 and β-actin in apoVs and MSCs. A-B light field image(A) and exposure image(B) of caspase-3, cleaved caspase-3 and β-actin.
